# The Selective SIK2 Inhibitor ARN-3236 Produces Strong Antidepressant-Like Efficacy in Mice via the Hippocampal CRTC1-CREB-BDNF Pathway

**DOI:** 10.3389/fphar.2020.624429

**Published:** 2021-01-14

**Authors:** Yue Liu, Wenqian Tang, Chunhui Ji, Jianghong Gu, Yanmei Chen, Jie Huang, Xinyi Zhao, Yingfang Sun, Chengniu Wang, Wei Guan, Jianfeng Liu, Bo Jiang

**Affiliations:** ^1^ Department of Pharmacology, School of Pharmacy, Nantong University, Nantong, China; ^2^ Provincial Key Laboratory of Inflammation and Molecular Drug Target, Nantong, China; ^3^ Basic Medical Research Centre, Medical College, Nantong University, Nantong, China

**Keywords:** ARN-3236, brain-derived neurotrophic factor, cyclic AMP response element binding protein, CREB-Regulated transcription coactivator 1, depression, hippocampus, salt-inducible kinase 2

## Abstract

Depression is a widespread chronic medical illness affecting thoughts, mood, and physical health. However, the limited and delayed therapeutic efficacy of monoaminergic drugs has led to intensive research efforts to develop novel antidepressants. ARN-3236 is the first potent and selective inhibitor of salt-inducible kinase 2 (SIK2). In this study, a multidisciplinary approach was used to explore the antidepressant-like actions of ARN-3236 in mice. Chronic social defeat stress (CSDS) and chronic unpredictable mild stress (CUMS) models of depression, various behavioral tests, high performance liquid chromatography-tandem mass spectrometry, stereotactic infusion, viral-mediated gene transfer, western blotting, co-immunoprecipitation and immunofluorescence were used together. It was found that ARN-3236 could penetrate the blood-brain barrier. Repeated ARN-3236 administration induced significant antidepressant-like effects in both the CSDS and CUMS models of depression, accompanied with fully preventing the stress-enhanced SIK2 expression and cytoplasmic translocation of cyclic adenosine monophosphate response element binding protein (CREB)-regulated transcription coactivator 1 (CRTC1) in the hippocampus. ARN-3236 treatment also completely reversed the down-regulating effects of CSDS and CUMS on the hippocampal brain-derived neurotrophic factor (BDNF) system and neurogenesis. Moreover, we demonstrated that the hippocampal CRTC1-CREB-BDNF pathway mediated the antidepressant-like efficacy of ARN-3236. Collectively, ARN-3236 possesses strong protecting effects against chronic stress, and could be a novel antidepressant beyond monoaminergic drugs.

## Highlights

1. ARN-3236 administration prevented both the CSDS-induced and CUMS-induced depressive-like behaviors in mice.
2. ARN-3236 administration antagonized the effects of both CSDS and CUMS on the hippocampal SIK2-CRTC1-CREB system.
3. ARN-3236 administration reversed the decreasing effects of both CSDS and CUMS on the hippocampal BDNF signaling cascade and neurogenesis.

## Introduction

As a widespread chronic medical illness affecting thoughts, mood, and physical health, depression is characterized by low mood, lack of energy, sadness, insomnia, and an inability to enjoy life (Martin et al., 2013; Ménard et al., 2016). However, so far the mechanisms of depression are vast and not fully understood, and as a result current treatments of patients with depression do not have a satisfactory therapeutic outcome (Krishnan and Nestler, 2008; Dean and Keshavan, 2017; Lima-Ojeda et al., 2018). Conventional antidepressants used in clinical practice include selective serotonin reuptake inhibitors (SSRIs: fluoxetine, escitalopram, etc.), serotonin and norepinephrine reuptake inhibitors (SNRIs: venlafaxine, duloxetine, etc.), norepinephrine-dopamine reuptake inhibitors (NDRIs: such as bupropion) and so on (López-Muñoz and Alamo, 2009; Pereira and Hiroaki-Sato, 2018). These antidepressants are based on the monoaminergic hypothesis of depression and have shown effectiveness in the treatment of major depression. However, data from clinical trials suggest that these drugs have response and remission rates of 60% and 40%, respectively (Alamo and López-Muñoz, 2009; Blier and El Mansari, 2013; Dale et al., 2015). Moreover, the delay in the appearance of the beneficial clinical response also limits the effectiveness of these therapies (Alamo and López-Muñoz, 2009; Blier and El Mansari, 2013; Dale et al., 2015). Although the blockade of monoamine reuptake is almost immediate, weeks of treatment is always needed to achieve a significant improvement in the depressive symptomatology. Nowadays it is thought that the onset of the therapeutic effects of monoaminergic antidepressants shall be the period in which crucial neurobiological adaptations occur to restore the brain's network activity (Vidal et al., 2011; Massart et al., 2012; Pilar-Cuéllar et al., 2013). Anyway, the limited and delayed therapeutic efficacy of monoaminergic drugs has led to intensive research efforts to develop novel antidepressants.

Salt-inducible kinases (SIKs, SIK1-3) are serine/threonine kinases which belong to the family of AMP-activated protein kinases (AMPKs), and have wide expression in many tissues such as adipocytes, hypothalamus, hippocampus, cerebral cortex and so on (Wang et al., 1999; Feldman et al., 2000; Katoh et al., 2004; Gallo and Iadecola, 2011; Jiang et al., 2019). SIKs have so far mainly been suggested to regulate gene expression by phosphorylating transcriptional regulators like the cAMP response element binding protein (CREB)-regulated transcription co-activators (CRTCs) (Takemori et al., 2007; Takemori and Okamoto, 2008; Choi et al., 2011; Gallo and Iadecola, 2011; Jiang et al., 2019). CRTCs, when phosphorylated, are sequestered in the cytoplasm where they are unable to activate the CREB-induced gene transcription (Altarejos and Montminy, 2011; Jurek et al., 2015; Rahnert et al., 2016). In contrast, CRTCs are dephosphorylated in response to elevations in cAMP, leading to nuclear translocation and binding to CREB (Altarejos and Montminy, 2011; Jurek et al., 2015; Rahnert et al., 2016). In 2019, we have reported that chronic stress-induced depression was accompanied by increased SIK2 expression and decreased nuclear CRTC1 translocation and CRTC1-CREB binding in the hippocampus (Jiang et al., 2019). Hippocampal SIK2 overexpression mimicked chronic stress that produced depressive-like phenotypes in naïve mice, whereas knockdown and knockout of hippocampal SIK2 protected against chronic stress (Jiang et al., 2019). Furthermore, the BDNF signaling cascade mediates the role of the hippocampal SIK2-CRTC1 system in the pathogenesis of depression (Jiang et al., 2019). We have also demonstrated that the actions of fluoxetine, venlafaxine and mirtazapine all involve the hippocampal SIK2-CRTC1 system (Jiang et al., 2019). Thus, hippocampal SIK2 could be a novel target for antidepressant developments.

ARN-3236 is the first potent, orally active and selective inhibitor of SIK2, with IC50s of <1 nM, 21.63 nM and 6.63 nM for SIK2, SIK1 and SIK3, respectively. However, so far there are few reports involving the pharmacological effects of ARN-3236. Here we speculated that ARN-3236 may have antidepressant-like actions due to SIK2 inhibition. In this study, a multidisciplinary approach was used to explore our assumption.

## Methods and Materials

### Ethical Statements

The experimental procedures involving animals and their care were conducted in compliance with the ARRIVE guidelines (Kilkenny et al., 2010; McGrath and Lilley, 2015), and approved by the Animal Welfare Committee of Nantong University. All efforts were made to minimize animal suffering.

### Animals

Adult C57BL/6J mice (male, 8 weeks old, 22–24 g) and CD1 mice (male and female, 50 weeks old) were bought from SLAC Laboratory Animal Co., Ltd. (Shanghai, China). C57BL/6J mice were the experimental subjects and subjected to stratified randomization according to body weight. Before use, all C57BL/6J mice were acclimatized to standard housing for 1 week with *ad libitum* access to water and rodent chow, as we previously described (Jiang et al., 2019). The behavioral testing was conducted between 8:00 am to 5:00 pm, and afterward, C57BL/6J mice were randomly selected and sacrificed at 9:00 am for all *in vitro* studies. The sample sizes were determined by power analysis (Unpaired two-tailed T-test, 95% confidence, 80% power) and according to our previous reports (Song et al., 2018; Jiang et al., 2019). For behavioral assays, each experimental group consisted of 10 mice. For biochemical assays, each experimental group consisted of five mice. A total 1,104 of experimental C57BL/6J mice were used in this study. All the behavioral tests were conducted in a blinded manner.

## Materials

Fluoxetine and ARN-3236 (Molecular Weight: 336.41) were obtained from Target Mol (Boston, United States; Cat# T0450L) and MedKoo Biosciences (Morrisville, USA; Cat# 206832), respectively. For intraperitoneal injection (i.p., 10 ml/kg) of fluoxetine/ARN-3236, the vehicle was 5% DMSO + 95% diluents (30% SBE-β-CD in 0.9% saline). For hippocampal infusion of ARN-3236, the vehicle was 5% DMSO + 95% diluents (30% SBE-β-CD in ACSF). 5-bromo-2-deoxyuridine (Brdu) was bought from Sigma (St. Louis, USA; Cat# 19-160) and dissolved in 0.9% saline. The i.p. doses of fluoxetine (20 mg/kg), ARN-3236 (1, 3, 10, 30 and 60 mg/kg) and Brdu (75 mg/kg) were chosen based on previous reports (Zhou et al., 2017; Jiang et al., 2019). The stereotactic doses of ARN-3236 (1 and 2 nmol) were determined according to the HPLC-MS study.

### Chronic Social Defeat Stress (CSDS)

CSDS was done as previously described (Jiang et al., 2017; Wang et al., 2017; Song et al., 2018; Xu et al., 2018; Jiang et al., 2019). Enough amounts of aggressive male CD1 mice were selected according to different experimental designs. In brief, each experimental C57BL/6J mouse was exposed to a CD1 aggressor for up to 10 min. After the defeat session, the two mice were kept in the same cage but separated by a plastic separator with holes for the remainder of the day. This procedure was repeated for 10 consecutive days, using a different CD1 aggressor every day. The separators were set immediately when the C57BL/6J mice displayed signs of stress and subordination (immobility, crouching, trembling, fleeing and upright posture; usually 7–10 min). The control C57BL/6J mice were pair-housed and handled daily. After CSDS, the experimental C57BL/6J mice were individually housed, and administration of fluoxetine/ARN-3236/vehicle was performed daily for another 2 weeks. The forced swim test (FST), tail suspension test (TST), sucrose preference test (SPT) and social interaction test were used together to evaluate the CSDS-induced depressive symptomatology. After the behavioral tests, the experimental C57BL/6J mice were subjected to either biochemical studies or euthanasia (anesthetized using carbon dioxide and then sacrificed by cervical dislocation).

### Chronic Unpredictable Mild Stress (CUMS)

CUMS was done as previously described (Ren et al., 2017; Ni et al., 2018; Xu et al., 2018; Jiang et al., 2019; Zhang et al., 2019). In brief, the experimental C57BL/6J mice were housed individually and subjected daily to 8 weeks of CUMS exposure which consisted of a random combination of eight stressors, including food or water deprivation (23 h), damp sawdust (12 h), restraint (2 h), cage rotation (30 min), inversion of light/dark cycle, 45°C cage tilting in empty cage (12 h) and cold (4°C for 1 h). The control C57BL/6J mice were handled daily without CUMS. Administration of fluoxetine/ARN-3236/vehicle was performed daily during the last 2 weeks. The FST, TST and SPT were used together to evaluate the CUMS-induced depressive symptomatology. After the behavioral tests, the experimental C57BL/6J mice were subjected to either biochemical studies or euthanasia (anesthetized using carbon dioxide and then sacrificed by cervical dislocation).

### Forced Swim Test (FST)

FST was performed as previously used (Jiang et al., 2017; Ren et al., 2017; Wang et al., 2017; Ni et al., 2018; Song et al., 2018; Xu et al., 2018; Jiang et al., 2019; Zhang et al., 2019; Wang et al., 2020). Briefly, the experimental C57BL/6J mice were forced to swim individually in an clear cylinder (45 cm height, 20 cm internal diameter) filled with fresh water (25 ± 1°C, 15 cm depth) for 6 min. Testing was videotaped and scored by an investigator unaware of animal grouping. The duration of immobility (floating in the water with only movements necessary to keep the nose above the surface) was recorded for the last 4 min. The water was changed after each trial.

### Tail Suspension Test (TST)

TST was performed as previously used (Jiang et al., 2017; Ren et al., 2017; Wang et al., 2017; Ni et al., 2018; Song et al., 2018; Xu et al., 2018; Jiang et al., 2019; Zhang et al., 2019; Wang et al., 2020) and lasted for 6 min. In brief, it was done by individually suspending the experimental C57BL/6J mice 60 cm above floor using adhesive tape placed to the tail 2 cm from the tip. A clear hollow climbstopper cylinder (4 cm length, 1.5 cm diameter) was placed around the tail to prevent tail-climbing behavior. Testing was videotaped and scored by an investigator unaware of animal grouping. The duration of immobility (when they hung passively and were completely motionless) was recorded for the whole 6-min period.

### Sucrose Preference Test (SPT)

This test was conducted as previously used (Jiang et al., 2017; Ren et al., 2017; Wang et al., 2017; Ni et al., 2018; Song et al., 2018; Xu et al., 2018; Jiang et al., 2019; Zhang et al., 2019; Wang et al., 2020). In brief, the experimental C57BL/6J mice were habituated to two bottles (one with 1% sucrose solution and the other with fresh water) for 48 h, and the bottle positions were counterbalanced across days (every 6 h). Afterward, the animals were deprived of water and food for 18 h, and then given pre-weighed bottles of 1% sucrose and water for 6 h testing (bottle positions interchanged every 2 h). The sucrose preference index was defined as the ratio of sucrose solution consumed vs. total liquid consumed.

### Social Interaction Test

This test was conducted as previously used (Jiang et al., 2017; Wang et al., 2017; Song et al., 2018; Xu et al., 2018; Jiang et al., 2019; Wang et al., 2020). It is a two-step procedure. In the first 5-min session (target absent), the experimental C57BL/6J mice were individually allowed to freely explore an open-field area (50 × 50 × 45 cm) which contained an empty circular wire cage (9 cm diameter) along one side. In the second 5-min session (target present), the experimental C57BL/6 mice were individually reintroduced into this arena now which contained a social target (unfamiliar male CD1 mouse) within the wire cage. The duration of time spent in the interaction zone (around the wire cage, 14 × 26 cm) for each session was recorded using an automated video tracking system (XinRuan Information Technology Co., Ltd., Shanghai, China). The open-field area was cleaned after each session.

### Statistical Analysis

Data were subjected to statistical analyses with SPSS 13.0 software (SPSS Inc., Chicago, United States) and represented as means ± S.E.M. Multiple group comparisons were performed using two-way ANONA + Bonferroni's test, or one-way ANONA + Tukey's test. Statistical significance was determined at *p* < 0.05.

### Additional Methods and Materials

See the Supplementary Material for description of high performance liquid chromatography-tandem mass spectrometry (HPLC-MS), stereotactic surgery and infusion, adenovirus associated virus (AAV)-mediated gene interference, co-immunoprecipitation (Co-IP) and other details.

## Results

### Intraperitoneal Injection of ARN-3236 Produced Significant Antidepressant-like Effects in Both the CSDS and CUMS Models of Depression

**FIGURE 1:**
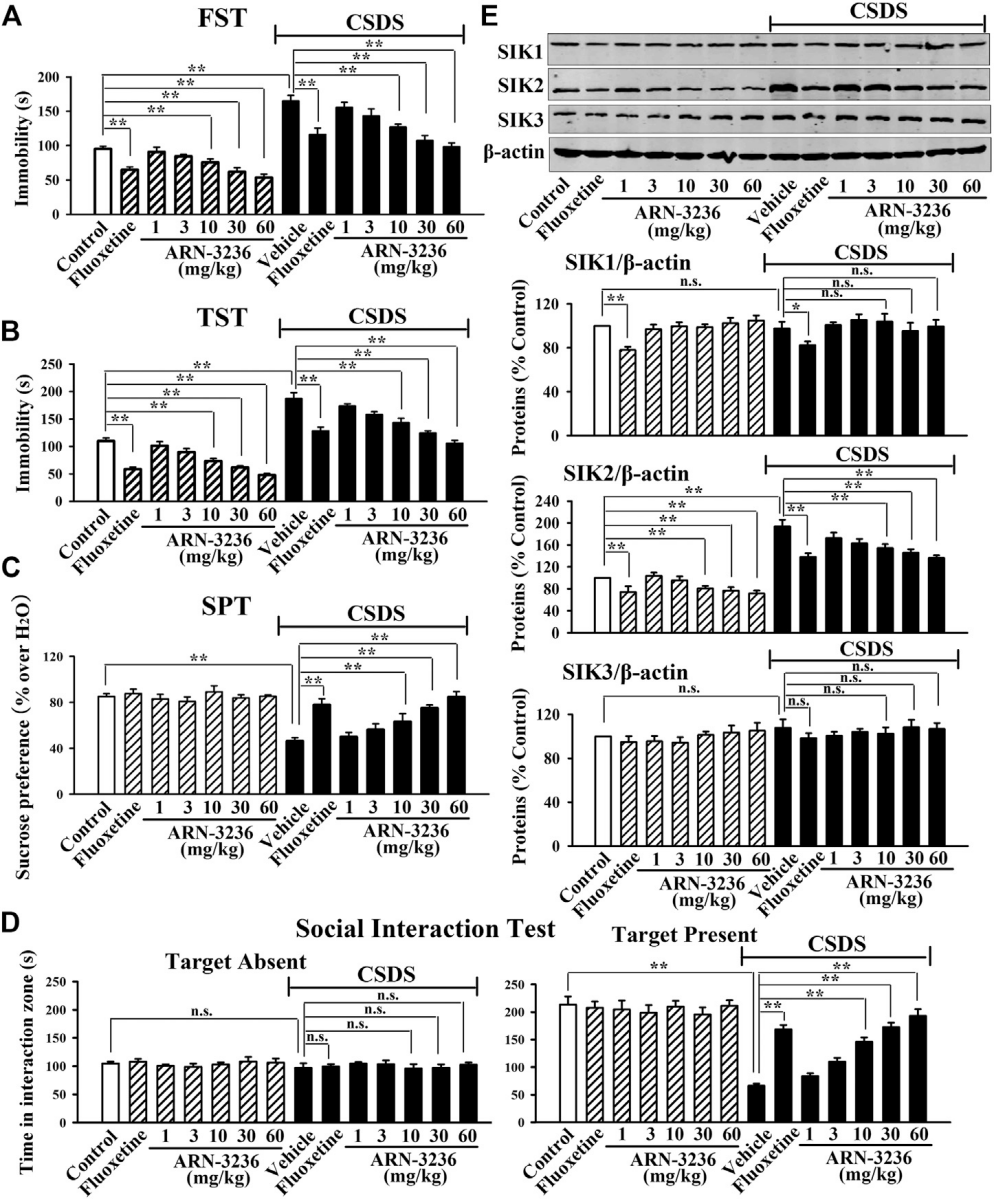
Intraperitoneal injection of ARN-3236 produced significant antidepressant-like effects in the CSDS model of depression, as revealed by the FST **(A)**, TST **(B)**, SPT **(C)** and social interaction test **(D)**. **(E)** Representative western blotting images and quantitative analyses show the effects of CSDS and ARN-3236 on hippocampal SIK1-3. All results were represented as means ± S.E.M (*n* = 10 for A-D, *n* = 5 for E); **p < 0.01; n.s., no significance. The comparisons were made by two-way ANOVA followed by Bonferroni's test.

**FIGURE 2:**
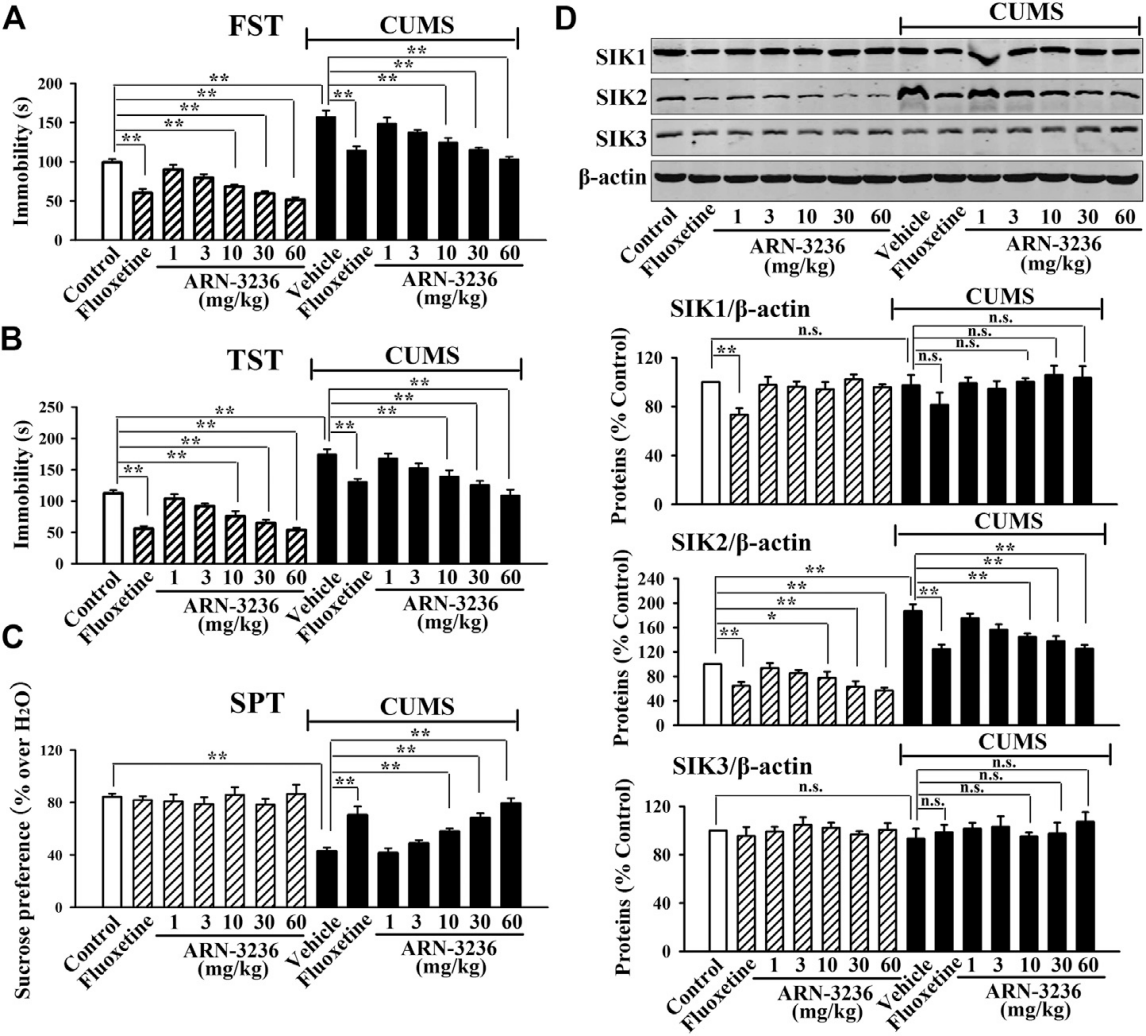
Intraperitoneal injection of ARN-3236 produced significant antidepressant-like effects in the CUMS model of depression, as revealed by the FST **(A)**, TST **(B)** and SPT **(C)**. **(D)** Representative western blotting images and quantitative analyses show the effects of CUMS and ARN-3236 on hippocampal SIK1-3. All results were represented as means ± S.E.M (*n* = 10 for A-C, *n* = 5 for **D**); **p < 0.01; n.s., no significance. The comparisons were made by two-way ANOVA followed by Bonferroni's test.

The possible antidepressant-like effects of ARN-3236 were first examined in both CSDS and CUMS, two well-validated models of depression, with fluoxetine used as the positive control (Forbes et al., 1996; Berton et al., 2006). As shown in Figures 1A–D–1D, 2A–C–2C, compared to the vehicle-treated control group, the CSDS-treated and CUMS-treated mice exhibited significantly more immobility in the FST and TST (helplessness), decreased sucrose preference (anhedonia) and less social interaction (social avoidance), revealing notable depressive-like behaviors (*n* = 10). In contrast, i.p. treatment of fluoxetine and ARN-3236 fully reversed these behavioral changes (*n* = 10). Detailed analyses showed that ARN-3236 produced a dose-dependent antidepressant action in mice between 10 and 60 mg/kg, and the effects of 30 and 60 mg/kg ARN-3236 were comparable and slightly superior to that of 20 mg/kg fluoxetine, respectively. In addition, i.p. treatment of fluoxetine and ARN-3236 also reduced the immobility of naïve control mice in the FST and TST (*n* = 10).

Subsequently, western blotting analyses were done to examine hippocampal SIK1-3 between all groups. As shown in Figures 1E, 2D, both CSDS and CUMS largely elevated the expression of hippocampal SIK2 (*n* = 5), but produced none effects on hippocampal SIK1 and SIK3 (*n* = 5), consistent with our previous report (Jiang et al., 2019). It was found that fluoxetine and ARN-3236 not only fully antagonized the promoting effects of chronic stress on the hippocampal SIK2 expression (*n* = 5), but also down-regulated such level in naïve control mice (*n* = 5), in parallel with the above behavioral results. Like CSDS and CUMS, ARN-3236 had none effects on hippocampal SIK1 and SIK3 (*n* = 5), proving its pharmacological specificity. Fluoxetine did not influence hippocampal SIK3 (*n* = 5), but had down-regulating actions on hippocampal SIK1 (*n* = 5).

### Hippocampal Infusion of ARN-3236 Protected Against Both CSDS and CUMS in Mice

To confirm whether ARN-3236 could cross the blood-brain barrier (BBB), the HPLC-MS method was adopted. As shown in Supplementary Figures S1 and S2, at 1 h after a single injection of 30 mg/kg ARN-3236, 309.79 ± 62.46 ng (approximately 1 nmol) ARN-3236 was detected in the hippocampus tissues of mice (*n* = 12). Also, at 2 h after drug administration, 293.69 ± 43.17 ng (approximately 1 nmol) ARN-3236 was detected in the hippocampus tissues of mice (*n* = 11). Thus, ARN-3236 is able to penetrate BBB.

**FIGURE 3:**
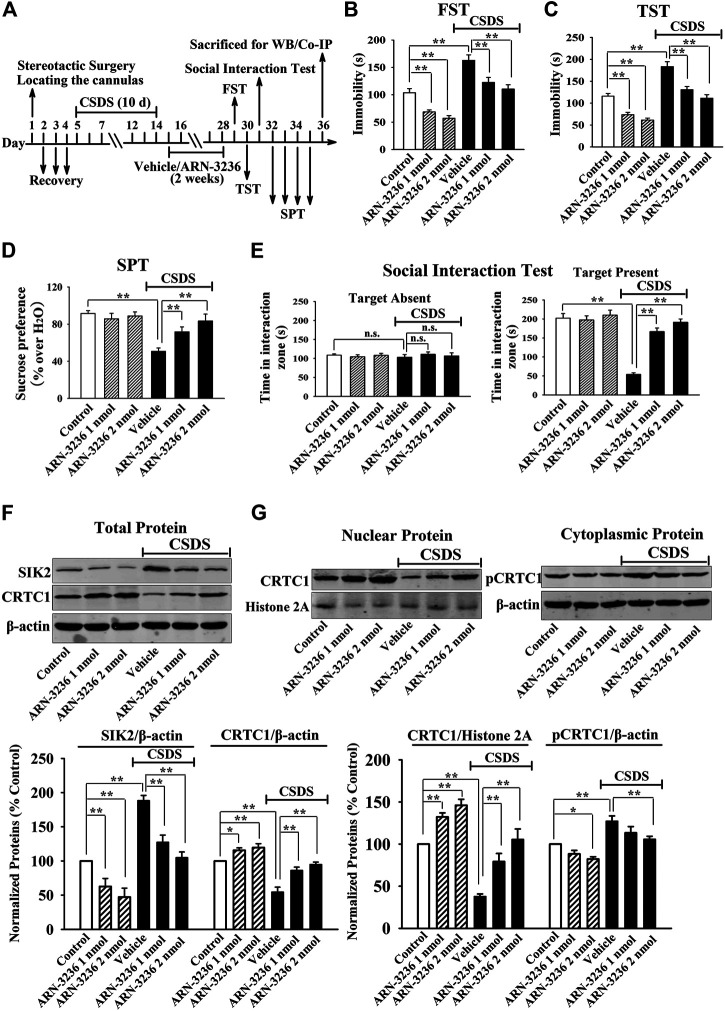
Hippocampal infusion of ARN-3236 induced notable antidepressant-like actions in the CSDS model of depression, as revealed by the FST **(B)**, TST **(C)**, SPT **(D)** and social interaction test **(E)**. Schematic timeline of the experimental procedures is shown in **(A)**. **(F)** Representative western blotting images and quantitative analyses show the effects of CSDS and ARN-3236 on hippocampal SIK2 and total CRTC1. **(G)** Representative western blotting images and quantitative analyses show the effects of CSDS and ARN-3236 on nuclear CRTC1 and cytoplasmic pCRTC1 in the hippocampus. All results were represented as means ± S.E.M (*n* = 10 for B–E, *n* = 5 for F–G); * *p* < 0.05, **p < 0.01; n.s., no significance. The comparisons were made by two-way ANOVA followed by Bonferroni's test.

**FIGURE 4:**
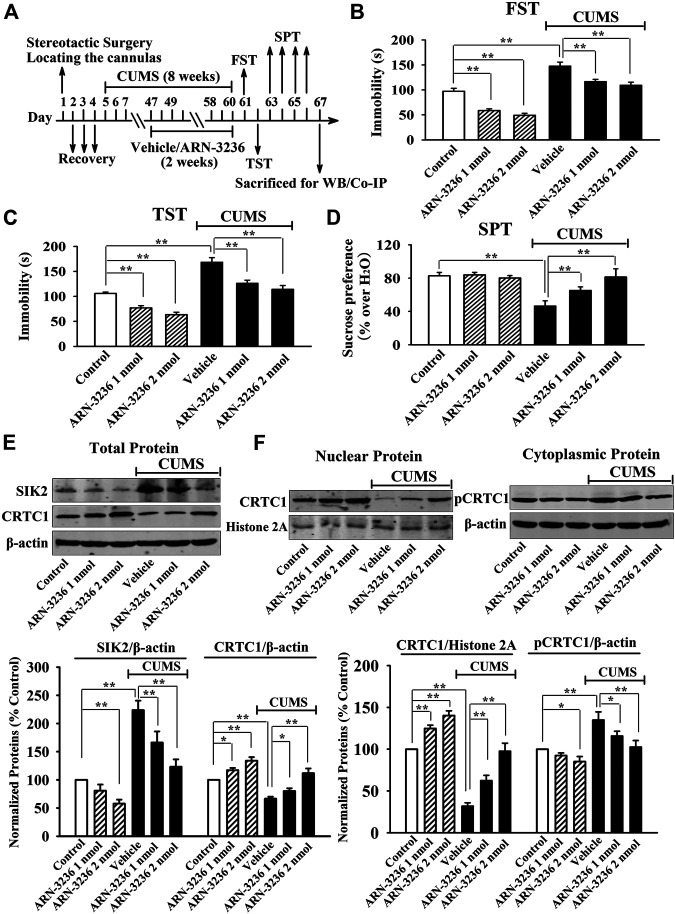
Hippocampal infusion of ARN-3236 induced notable antidepressant-like actions in the CUMS model of depression, as revealed by the FST **(B)**, TST **(C)** and SPT **(D)**. Schematic timeline of the experimental procedures is shown in **(A)**. **(E)** Representative western blotting images and quantitative analyses show the effects of CUMS and ARN-3236 on hippocampal SIK2 and total CRTC1. **(F)** Representative western blotting images and quantitative analyses show the effects of CUMS and ARN-3236 on nuclear CRTC1 and cytoplasmic pCRTC1 in the hippocampus. All results were represented as means ± S.E.M (*n* = 10 for B–D, *n* = 5 for E–F**)**; * *p* < 0.05, **p < 0.01. The comparisons were made by two-way ANOVA followed by Bonferroni's test.

Next, 1 or 2 nmol ARN-3236 was stereotactically infused into the hippocampus of the CSDS-treated and CUMS-treated mice. Figures 3B–E indicated that the (CSDS + 1 nmol ARN-3236)-treated and (CSDS + 2 nmol ARN-3236)-treated mice all had significantly increased sucrose preference [ANOVA: CSDS, F(1, 54) = 20.174, *p* < 0.01; ARN-3236, F(2, 54) = 16.405, *p* < 0.01; Interaction, F(2, 54) = 12.694, *p* < 0.01] and social interaction [ANOVA: CSDS, F(1, 54) = 42.359, *p* < 0.01; ARN-3236, F(2, 54) = 33.601, *p* < 0.01; Interaction, F(2, 54) = 27.668, *p* < 0.01], as well as reduced immobility in the FST [ANOVA: CSDS, F(1, 54) = 28.157, *p* < 0.01; ARN-3236, F(2, 54) = 22.679, *p* < 0.01; Interaction, F(2, 54) = 18.498, *p* < 0.01] and TST [ANOVA: CSDS, F(1, 54) = 32.702, *p* < 0.01; ARN-3236, F(2, 54) = 20.661, *p* < 0.01; Interaction, F(2, 54) = 24.487, *p* < 0.01], than those of the CSDS-treated mice (*n* = 10). Figure 4B–D indicated that the (CUMS + 1 nmol ARN-3236)-treated and (CUMS + 2 nmol ARN-3236)-treated mice also displayed evidently higher sucrose preference [ANOVA: CUMS, F(1, 54) = 19.728, *p* < 0.01; ARN-3236, F(2, 54) = 13.796, *p* < 0.01; Interaction, F(2, 54) = 17.435, *p* < 0.01] and less immobility in the FST [ANOVA: CUMS, F(1, 54) = 25.224, *p* < 0.01; ARN-3236, F(2, 54) = 15.725, *p* < 0.01; Interaction, F(2, 54) = 19.046, *p* < 0.01] and TST [ANOVA: CUMS, F(1, 54) = 29.346, *p* < 0.01; ARN-3236, F(2, 54) = 21.823, *p* < 0.01; Interaction, F(2, 54) = 23.626, *p* < 0.01] than those of the CUMS-treated mice (*n* = 10). Besides, hippocampal infusion of ARN-3236 significantly decreased the immobility of naïve control mice in the FST and TST (*n* = 10). All these data are in accordance with the i.p. behavioral results.

**FIGURE 5:**
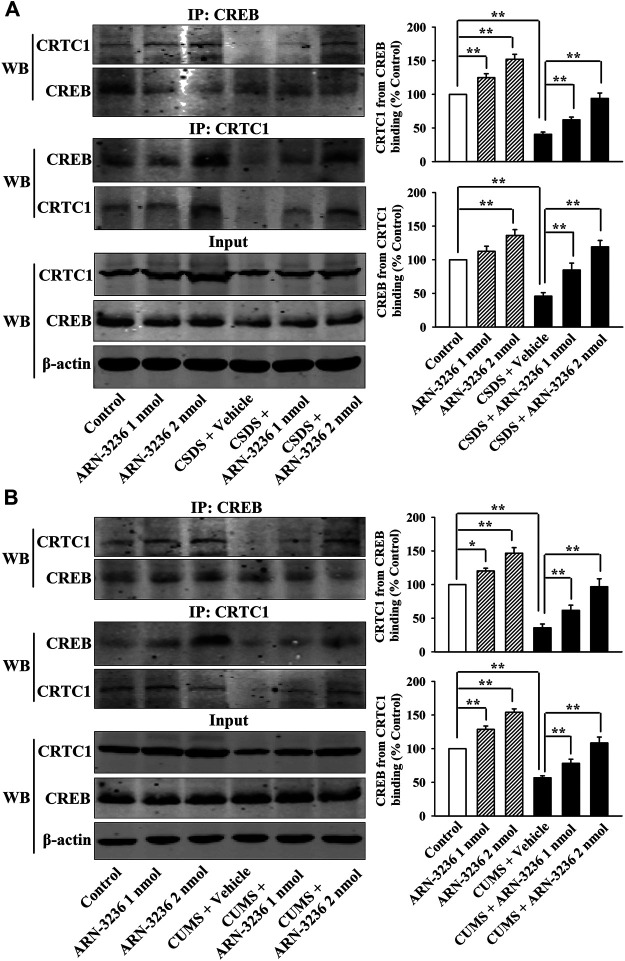
Hippocampal infusion of ARN-3236 fully prevented the down-regulating effects of chronic stress on CRTC1-CREB binding in the hippocampus. **(A)** Representative Co-IP images and quantitative analyses show the effects of CSDS and ARN-3236 on hippocampal CRTC1-CREB binding. **(B)** Representative Co-IP images and quantitative analyses show the effects of CUMS and ARN-3236 on hippocampal CRTC1-CREB binding. All results were represented as means ± S.E.M (*n* = 5); *p < 0.05, **p < 0.01. The comparisons were made by two-way ANOVA followed by Bonferroni's test.

Afterward, the hippocampal SIK2-CRTC1 signaling changes of all groups were detected. It was found that ARN-3236 infusion not only fully restored the CSDS-induced increase in hippocampal SIK2 [ANOVA: CSDS, F(1, 24) = 37.186, *p* < 0.01; ARN-3236, F(2, 24) = 28.223, *p* < 0.01; Interaction, F(2, 24) = 24.775, *p* < 0.01] (Figure 3F, *n* = 5) and cytoplasmic pCRTC1 [ANOVA: CSDS, F(1, 24) = 21.492, *p* < 0.01; ARN-3236, F(2, 24) = 14.228, *p* < 0.01; Interaction, F(2, 24) = 11.502, *p* < 0.01] (Figure 3G, *n* = 5) expression, but also notably reversed the CSDS-induced decrease in hippocampal levels of total CRTC1 [ANOVA: CSDS, F(1, 24) = 27.559, *p* < 0.01; ARN-3236, F(2, 24) = 22.354, *p* < 0.01; Interaction, F(2, 24) = 18.613, *p* < 0.01] (Figure 3F, *n* = 5), nuclear CRTC1 [ANOVA: CSDS, F(1, 24) = 40.119, *p* < 0.01; ARN-3236, F(2, 24) = 32.008, *p* < 0.01; Interaction, F(2, 24) = 25.862, *p* < 0.01] (Figure 3G, *n* = 5) and CRTC1-CREB binding [ANOVA: CSDS, F(1, 24) = 36.073, *p* < 0.01; ARN-3236, F(2, 24) = 24.625, *p* < 0.01; Interaction, F(2, 24) = 28.339, *p* < 0.01] (Figure 5A, *n* = 5). Similarly, ARN-3236 infusion significantly prevented the CUMS-induced effects on hippocampal SIK2 [ANOVA: CUMS, F(1, 24) = 45.278, *p* < 0.01; ARN-3236, F(2, 24) = 35.721, *p* < 0.01; Interaction, F(2, 24) = 27.084, *p* < 0.01] (Figure 4E, *n* = 5), total CRTC1 [ANOVA: CUMS, F(1, 24) = 23.235, *p* < 0.01; ARN-3236, F(2, 24) = 17.075, *p* < 0.01; Interaction, F(2, 24) = 15.247, *p* < 0.01] (Figure 4E, *n* = 5), nuclear CRTC1 [ANOVA: CUMS, F(1, 24) = 38.362, *p* < 0.01; ARN-3236, F(2, 24) = 32.118, *p* < 0.01; Interaction, F(2, 24) = 25.071, *p* < 0.01] (Figure 4F, *n* = 5), cytoplasmic pCRTC1 [ANOVA: CUMS, F(1, 24) = 29.537, *p* < 0.01; ARN-3236, F(2, 24) = 24.081, *p* < 0.01; Interaction, F(2, 24) = 18.627, *p* < 0.01] (Figure 4F, *n* = 5) and CRTC1-CREB binding [ANOVA: CUMS, F(1, 24) = 31.343, *p* < 0.01; ARN-3236, F(2, 24) = 22.671, *p* < 0.01; Interaction, F(2, 24) = 25.652, *p* < 0.01] (Figure 5B, *n* = 5). Moreover, ARN-3236 infusion down-regulated the level of cytoplasmic pCRTC1 (Figures 3G, 4F, *n* = 5) and up-regulated the levels of total CRTC1 (Figures 3F, 4E, *n* = 5), nuclear CRTC1 (Figures 3G, 4F, *n* = 5) and CRTC1-CREB binding (Figure 5, *n* = 5) in the hippocampus of naive control mice.

**FIGURE 6:**
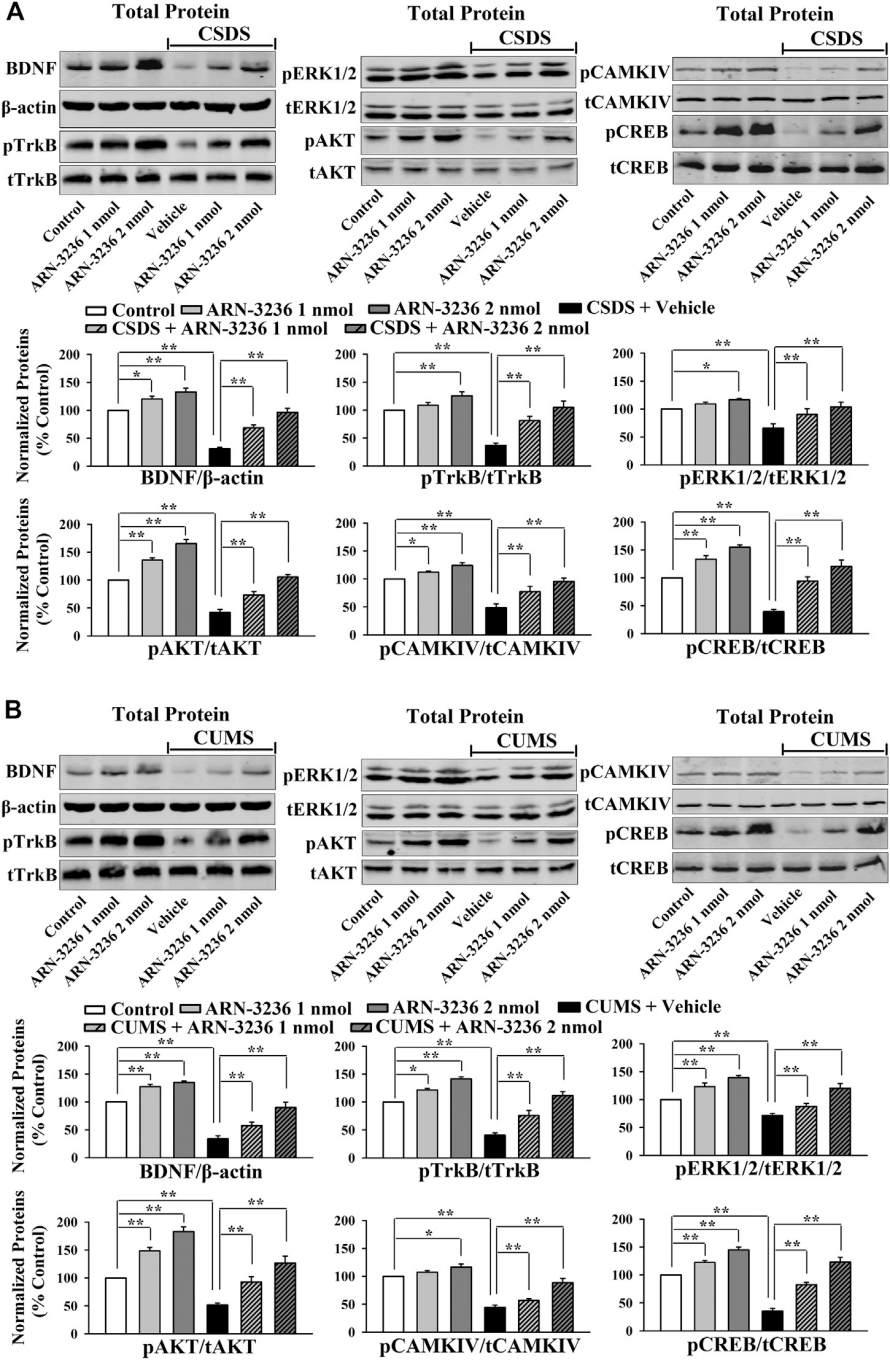
Hippocampal infusion of ARN-3236 protected against the decreasing effects of chronic stress on the hippocampal BDNF system. **(A)** Representative western blotting images and quantitative analyses show the effects of CSDS and ARN-3236 on the expression of the hippocampal BDNF-TrkB-ERK1/2/AKT/CaMKIV-CREB pathway. **(B)** Representative western blotting images and quantitative analyses show the effects of CUMS and ARN-3236 on the level of the hippocampal BDNF-TrkB-ERK1/2/AKT/CaMKIV-CREB pathway. All results were represented as means ± S.E.M (*n* = 5); *p < 0.05, **p < 0.01. The comparisons were made by two-way ANOVA followed by Bonferroni's test.

**FIGURE 7:**
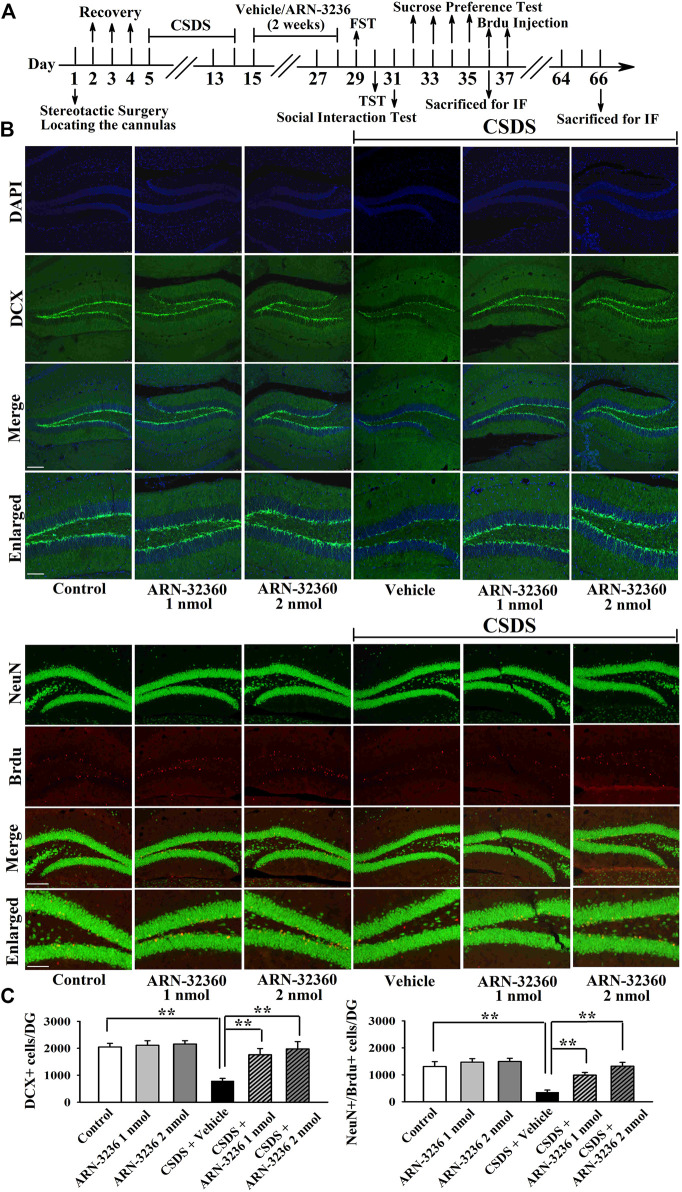
Hippocampal infusion of ARN-3236 markedly ameliorated the reducing effects of CSDS on hippocampal neurogenesis. **(A)** Schematic timeline of the experimental procedures. **(B)** Representative confocal and fluorescence microscopic images show the staining of DCX (green) and co-staining (yellow) of NeuN (green)/Brdu (red) in DG, respectively. Scale bar: 150 µm for representative images and 75 µm for enlarged images. **(C)** Quantitative analyses indicate that ARN-3236 treatment substantially increased the number of DCX^+^ cells and NeuN^+^/Brdu^+^ cells in DG of the CSDS-stressed mice. All results were represented as means ± S.E.M (*n* = 5); **p < 0.01. The comparisons were made by two-way ANOVA followed by Bonferroni's test.

**FIGURE 8:**
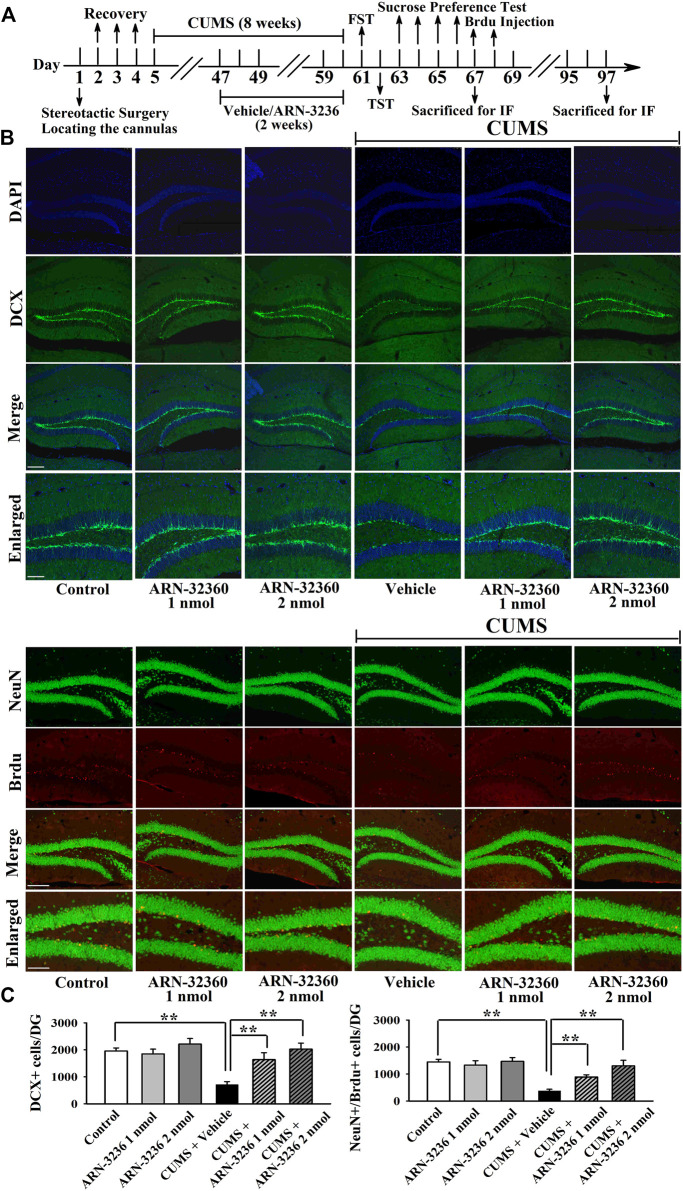
Hippocampal infusion of ARN-3236 markedly ameliorated the reducing effects of CUMS on hippocampal neurogenesis. **(A)** Schematic timeline of the experimental procedures. **(B)** Representative confocal and fluorescence microscopic images show the staining of DCX (green) and co-staining (yellow) of NeuN (green)/Brdu (red) in DG, respectively. Scale bar: 150 µm for representative images and 75 µm for enlarged images. **(C)** Quantitative analyses indicate that ARN-3236 treatment largely enhanced the number of DCX^+^ cells and NeuN^+^/Brdu^+^ cells in DG of the CUMS-stressed mice. All results were represented as means ± S.E.M (*n* = 5); **p < 0.01. The comparisons were made by two-way ANOVA followed by Bonferroni's test.

The hippocampal BDNF signaling cascade and neurogenesis among all groups were also examined. ARN-3236 infusion not only blocked the CSDS-induced and CUMS-induced decrease in the hippocampal BDNF [ANOVA: CSDS, F(1, 24) = 33.167, *p* < 0.01; ARN-3236, F(2, 24) = 27.886, *p* < 0.01; Interaction, F(2, 24) = 21.075, *p* < 0.01. CUMS, F(1, 24) = 28.395, *p* < 0.01; ARN-3236, F(2, 24) = 24.432, *p* < 0.01; Interaction, F(2, 24) = 18.255, *p* < 0.01], pTrkB [ANOVA: CSDS, F(1, 24) = 24.309, *p* < 0.01; ARN-3236, F(2, 24) = 16.824, *p* < 0.01; Interaction, F(2, 24) = 19.032, *p* < 0.01. CUMS, F(1, 24) = 26.114, *p* < 0.01; ARN-3236, F(2, 24) = 20.626, *p* < 0.01; Interaction, F(2, 24) = 15.439, *p* < 0.01], pERK1/2 [ANOVA: CSDS, F(1, 24) = 17.385, *p* < 0.01; ARN-3236, F(2, 24) = 12.613, *p* < 0.01; Interaction, F(2, 24) = 14.371, *p* < 0.01. CUMS, F(1, 24) = 19.608, *p* < 0.01; ARN-3236, F(2, 24) = 16.445, *p* < 0.01; Interaction, F(2, 24) = 13.072, *p* < 0.01], pAKT [ANOVA: CSDS, F(1, 24) = 39.471, *p* < 0.01; ARN-3236, F(2, 24) = 28.762, *p* < 0.01; Interaction, F(2, 24) = 33.116, *p* < 0.01. CUMS, F(1, 24) = 37.811, *p* < 0.01; ARN-3236, F(2, 24) = 30.504, *p* < 0.01; Interaction, F(2, 24) = 23.748, *p* < 0.01], pCaMKIV [ANOVA: CSDS, F(1, 24) = 26.835, *p* < 0.01; ARN-3236, F(2, 24) = 18.908, *p* < 0.01; Interaction, F(2, 24) = 16.459, *p* < 0.01. CUMS, F(1, 24) = 21.009, *p* < 0.01; ARN-3236, F(2, 24) = 12.288, *p* < 0.01; Interaction, F(2, 24) = 13.107, *p* < 0.01] and pCREB [ANOVA: CSDS, F(1, 24) = 34.992, *p* < 0.01; ARN-3236, F(2, 24) = 27.967, *p* < 0.01; Interaction, F(2, 24) = 25.559, *p* < 0.01. CUMS, F(1, 24) = 38.102, *p* < 0.01; ARN-3236, F(2, 24) = 33.306, *p* < 0.01; Interaction, F(2, 24) = 24.036, *p* < 0.01] expression, but also enhanced these proteins in naive control mice (Figure 6, *n* = 5). In contrast, the levels of total β-actin, TrkB, ERK1/2, AKT, CaMKIV and CREB in the hippocampus were unchanged among all groups (*n* = 5). Similarly, ARN-3236 infusion markedly prevented the CSDS-induced [ANOVA for DCX/DAPI: CSDS, F(1, 24) = 39.808, *p* < 0.01; ARN-3236, F(2, 24) = 34.066, *p* < 0.01; Interaction, F(2, 24) = 27.849, *p* < 0.01. ANOVA for Brdu/NeuN: CSDS, F(1, 24) = 41.314, *p* < 0.01; ARN-3236, F(2, 24) = 31.226, *p* < 0.01; Interaction, F(2, 24) = 28.195, *p* < 0.01] and CUMS-induced [ANOVA for DCX/DAPI: CUMS, F(1, 24) = 37.678, *p* < 0.01; ARN-3236, F(2, 24) = 29.838, *p* < 0.01; Interaction, F(2, 24) = 25.407, *p* < 0.01. ANOVA for Brdu/NeuN: CUMS, F(1, 24) = 34.075, *p* < 0.01; ARN-3236, F(2, 24) = 27.309, *p* < 0.01; Interaction, F(2, 24) = 20.783, *p* < 0.01] decrease in hippocampal neurogenesis (Figures 7, 8, *n* = 5). However, ARN-3236 infusion did not influence the hippocampal neurogenesis in naïve control mice (*n* = 5). Taken together, ARN-3236 possesses excellent antidepressant-like actions against chronic stress in mice.

### The Hippocampal CRTC1-CREB Pathway Mediates the Antidepressant-like Efficacy of ARN-3236

**FIGURE 9:**
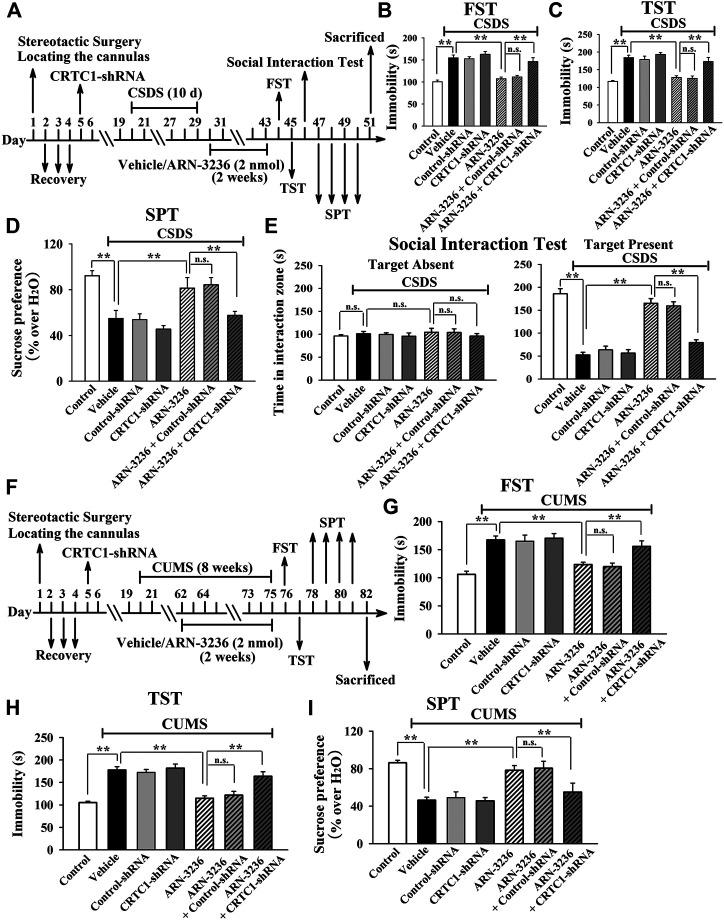
Hippocampal CRTC1-knockdown by using AAV-CRTC1-shRNA abolished the antidepressant-like effects of ARN-3236 in mice. **(A)** Schematic timeline of the CSDS-involved experimental procedures. **(B**–**E)** AAV-CRTC1-shRNA pre-infusion significantly prevented the reversal effects of ARN-3236 on the CSDS-induced depressive-like behaviors in the FST, TST, SPT and social interaction test. **(F)** Schematic timeline of the CUMS-involved experimental procedures. **(G**–**I)** AAV-CRTC1-shRNA pre-infusion fully blocked the restoring effects of ARN-3236 on the CUMS-induced depressive-like behaviors in the FST, TST and SPT. All results were represented as means ± S.E.M (*n* = 10); **p < 0.01; n.s., no significance. The comparisons were made by one-way ANOVA followed by Tukey's test.

To explore the antidepressant mechanism of ARN-3236, AAV-CRTC1-shRNA was first used, and its silencing efficacy has been confirmed in our previous study (Jiang et al., 2019). The CRTC1-shRNA-pretreated mice were subjected to CSDS and then infused with 2 nmol ARN-3236, followed by behavioral tests. As shown in Figures 9B–E, hippocampal CRTC1-knockdown fully abolished the antidepressant-like actions of ARN3236 against CSDS, as the (CSDS + ARN-3236 + CRTC1-shRNA)-treated mice displayed significantly decreased sucrose preference [ANOVA: F(6, 63) = 19.706, *p* < 0.01] and social interaction [ANOVA: F(6, 63) = 32.607, *p* < 0.01], as well as increased immobility in the FST [ANOVA: F(6, 63) = 23.388, *p* < 0.01] and TST [ANOVA: F(6, 63) = 27.109, *p* < 0.01], than those of the (CSDS + ARN-3236)-treated and (CSDS + ARN-3236 + Control-shRNA)-treated mice (*n* = 10). Moreover, the CRTC1-shRNA-pretreated mice were subjected to CUMS and 2 nmol ARN-3236 treatment, followed by behavioral tests. As shown in Figures 9G–I, hippocampal CRTC1-knockdown also prevented the antidepressant-like effects of ARN3236 against CUMS, as the (CUMS + ARN-3236 + CRTC1-shRNA)-treated mice exhibited evidently less sucrose preference [ANOVA: F(6, 63) = 20.439, *p* < 0.01] and more immobility in the FST [ANOVA: F(6, 63) = 17.505, *p* < 0.01] and TST [ANOVA: F(6, 63) = 25.112, *p* < 0.01] than those of the (CUMS + ARN-3236)-treated and (CUMS + ARN-3236 + Control-shRNA)-treated mice (*n* = 10).

**FIGURE 10:**
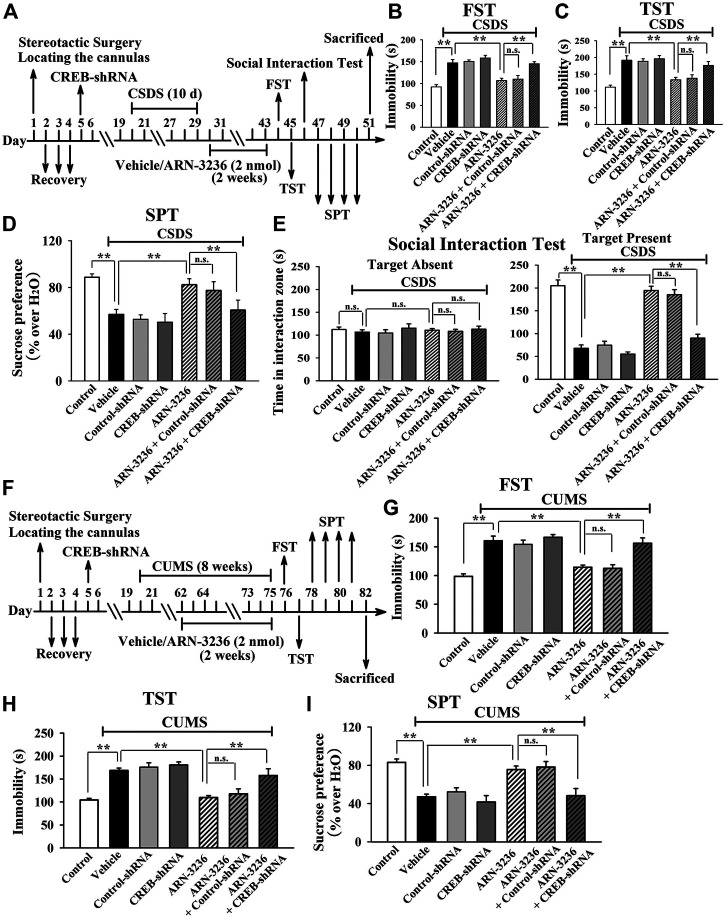
Hippocampal CREB-knockdown by using AAV-CREB-shRNA abolished the antidepressant-like effects of ARN-3236 in mice. **(A)** Schematic timeline of the CSDS-involved experimental procedures. **(B**–**E)** AAV-CREB-shRNA pre-infusion significantly prevented the reversal effects of ARN-3236 on the CSDS-induced depressive-like behaviors in the FST, TST, SPT and social interaction test. **(F)** Schematic timeline of the CUMS-involved experimental procedures. **(G**–**I)** AAV-CREB-shRNA pre-infusion fully blocked the restoring effects of ARN-3236 on the CUMS-induced depressive-like behaviors in the FST, TST and SPT. All results were represented as means ± S.E.M (*n* = 10); **p < 0.01; n.s., no significance. The comparisons were made by one-way ANOVA followed by Tukey's test.

Then, AAV–CREB–shRNA was used, and its silencing efficacy has been confirmed in our previous study (Jiang et al., 2019). The CREB–shRNA-pretreated mice were subjected to CSDS and then infused with 2 nmol ARN-3236, followed by behavioral tests. Figures 10B–E revealed that hippocampal CREB-knockdown fully blocked the antidepressant-like actions of ARN3236 against CSDS, as the (CSDS + ARN-3236 + CREB-shRNA)-treated mice displayed notably decreased sucrose preference [ANOVA: F(6, 63) = 22.563, *p* < 0.01] and social interaction [ANOVA: F(6, 63) = 44.108, *p* < 0.01], as well as increased immobility in the FST [ANOVA: F(6, 63) = 25.345, *p* < 0.01] and TST [ANOVA: F(6, 63) = 21.466, *p* < 0.01], than those of the (CSDS + ARN-3236)-treated and (CSDS + ARN-3236 + Control-shRNA)-treated mice (*n* = 10). In addition, the CREB-shRNA-pretreated mice were subjected to CUMS and 2 nmol ARN-3236 treatment, followed by behavioral tests. Figures 10G–I revealed that hippocampal CREB-knockdown also blocked the antidepressant-like effects of ARN3236 against CUMS, as the (CUMS + ARN-3236 + CREB-shRNA)-treated mice exhibited notably less sucrose preference [ANOVA: F(6, 63) = 16.503, *p* < 0.01] and more immobility in the FST [ANOVA: F(6, 63) = 22.919, *p* < 0.01] and TST [ANOVA: F(6, 63) = 19.206, *p* < 0.01] than those of the (CUMS + ARN-3236)-treated and (CUMS + ARN-3236 + Control-shRNA)-treated mice (*n* = 10).

Collectively, the antidepressant-like effects of ARN-3236 in mice require the hippocampal CRTC1-CREB pathway.

### The Hippocampal BDNF Signaling Cascade Is Necessary for the Antidepressant-like Efficacy of ARN-3236

**FIGURE 11:**
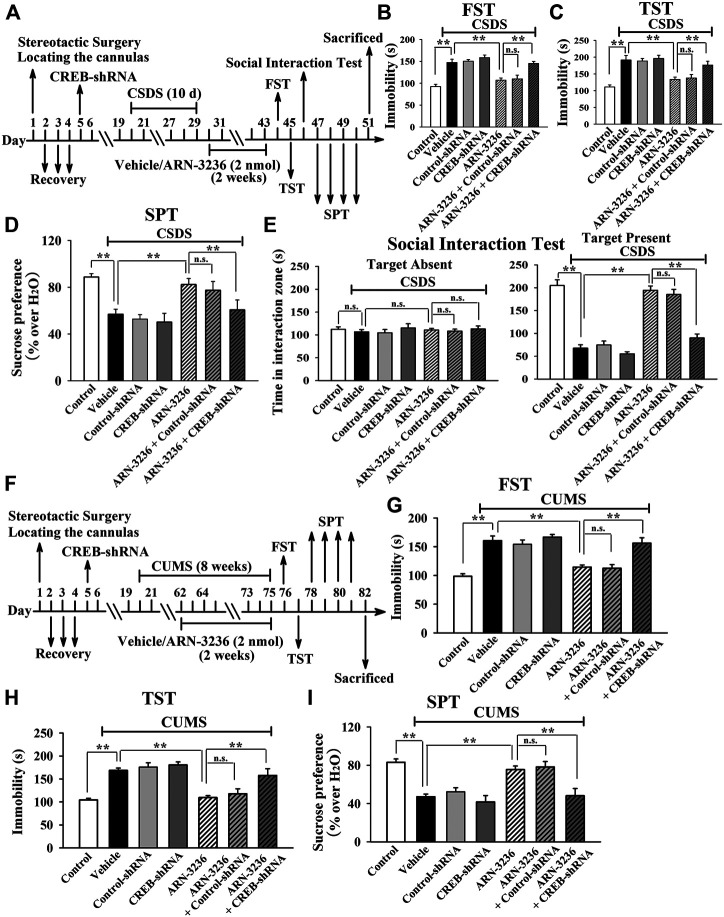
Hippocampal BDNF-knockdown by using AAV-BDNF-shRNA abrogated the ARN-3236-induced antidepressant-like actions in mice. **(A)** Schematic timeline of the CSDS-involved experimental procedures. **(B**–**E)** AAV-BDNF-shRNA pre-infusion markedly abolished the reversal effects of ARN-3236 on the CSDS-induced depressive-like behaviors in the FST, TST, SPT and social interaction test. **(F)** Schematic timeline of the CUMS-involved experimental procedures. **(G**–**I)** AAV-BDNF-shRNA pre-infusion notably antagonized the restoring effects of ARN-3236 on the CUMS-induced depressive-like behaviors in the FST, TST and SPT. All results were represented as means ± S.E.M (*n* = 10); **p < 0.01; n.s., no significance. The comparisons were made by one-way ANOVA followed by Tukey's test.

**FIGURE 12:**
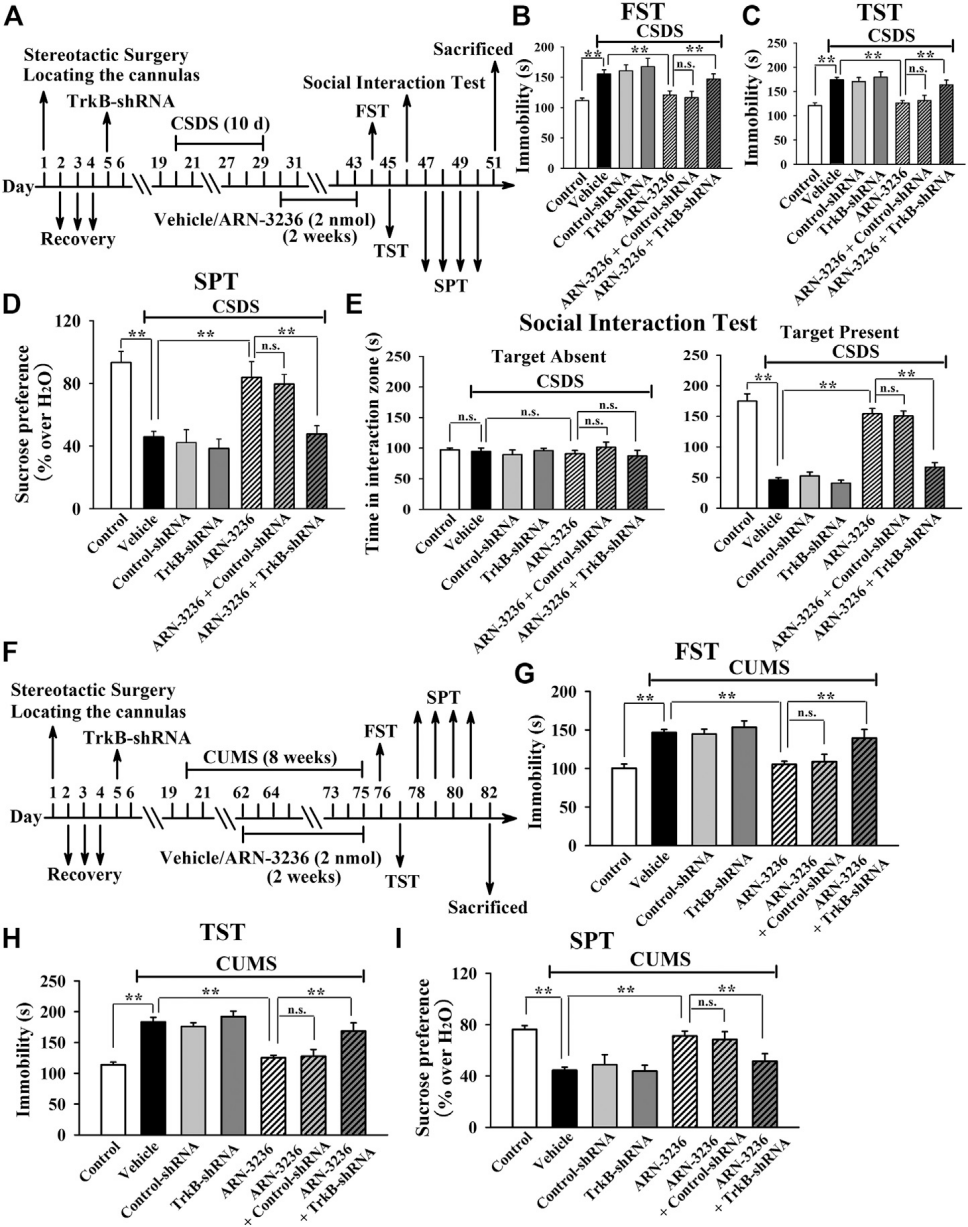
Hippocampal TrkB-knockdown by using AAV-TrkB-shRNA abrogated the ARN-3236-induced antidepressant-like actions in mice. **(A)** Schematic timeline of the CSDS-involved experimental procedures. **(B–E)** AAV-TrkB-shRNA pre-infusion markedly abolished the reversal effects of ARN-3236 on the CSDS-induced depressive-like behaviors in the FST, TST, SPT and social interaction test. **(F)** Schematic timeline of the CUMS-involved experimental procedures. **(G**–**I)** AAV-TrkB-shRNA pre-infusion notably antagonized the restoring effects of ARN-3236 on the CUMS-induced depressive-like behaviors in the FST, TST and SPT. All results were represented as means ± S.E.M (*n* = 10); **p < 0.01; n.s., no significance. The comparisons were made by one-way ANOVA followed by Tukey's test.

To further determine the downstream target underlying the antidepressant-like effects of ARN-3236, AAV-BDNF-shRNA was employed, and its silencing efficacy has been demonstrated before (Jiang et al., 2019). The BDNF-shRNA-pretreated mice were subjected to CSDS and then infused with 2 nmol ARN-3236, followed by behavioral tests. It was found that hippocampal BDNF-knockdown not only significantly prevented the ARN-32326-enhanced sucrose preference [ANOVA: F(6, 63) = 32.304, *p* < 0.01] and social interaction [ANOVA: F(6, 63) = 46.259, *p* < 0.01] in mice, but also markedly blocked the ARN-3236-reduced immobility of mice in the FST [ANOVA: F(6, 63) = 28.165, *p* < 0.01] and TST [ANOVA: F(6, 63) = 33.506, *p* < 0.01] (Figures 11B–E, *n* = 10). Similar results were observed in the CUMS model of depression [ANOVA for FST: F(6, 63) = 26.352, *p* < 0.01. ANOVA for TST: F(6, 63) = 29.206, *p* < 0.01. ANOVA for SPT: F(6, 63) = 24.918, *p* < 0.01] (Figures 11G–I, *n* = 10). Besides, AAV-TrkB-shRNA was also employed, and its silencing efficacy has been proved before (Jiang et al., 2019). The TrkB-shRNA-pretreated mice were subjected to CSDS and then infused with 2 nmol ARN-3236, followed by behavioral tests. Figures 12B–E showed that in parallel with the BDNF-shRNA data, hippocampal TrkB-knockdown notably abolished the protecting effects of ARN-3236 against CSDS in the FST [ANOVA: F(6, 63) = 28.859, *p* < 0.01], TST [ANOVA: F(6, 63) = 30.281, *p* < 0.01], SPT [ANOVA: F(6, 63) = 26.387, *p* < 0.01] and social interaction test [ANOVA: F(6, 63) = 38.604, *p* < 0.01] (*n* = 10). Similar results were observed again in the CUMS model of depression [ANOVA for FST: F(6, 63) = 24.661, *p* < 0.01. ANOVA for TST: F(6, 63) = 27.973, *p* < 0.01. ANOVA for SPT: F(6, 63) = 18.309, *p* < 0.01] (Figure 12G-12I, *n* = 10). Therefore, as well as CRTC1 and CREB, the BDNF system in the hippocampus also contributes to the antidepressant mechanism of ARN-3236.

## Discussion

To the best of our knowledge, our study is the first comprehensive study showing that ARN-3236 has beneficial effects against depression, a most burdensome neuropsychiatric disease worldwide. This finding is very interesting and meaningful as it has identified a new potential antidepressant beyond monoaminergic drugs. As the two most widely used and accepted model of depression in rodents, CSDS and CUMS can simulate many core symptoms of depression, including helplessness, anhedonia, social avoidance, decreased appetite, etc. (Yan et al., 2010; Chaouloff, 2013; Czéh et al., 2016; Antoniuk et al., 2019). The FST and TST have been widely used to detect potential antidepressant activities and to evaluate the helplessness behaviors of rodents (Cryan et al., 2005; Petit-Demouliere et al., 2005). Additionally, the sucrose preference test and social interaction test are employed to assess the anhedonia and social avoidance behaviors of rodents, respectively. The positive control, fluoxetine, displayed significant antidepressant actions in both the two models, indicated that our procedures were successful and reliable. Excitingly, it was found that i.p. injection of 30 mg/kg and 60 mg/kg ARN-3236 produced antidepressant efficacy in mice which was equal to or even better than that of 20 mg/kg fluoxetine.

ARN-3236 is chemically named 3-(2, 4-dimethoxyphenyl)-4-thiophen-3-yl-1H-pyrrolo[2,3-b]pyridine, possessing no hydrophilic groups and should therefore be liposoluble and able to penetrate BBB. As expected, the HPLC-MS results confirmed that ARN-3236 has good ability to cross BBB. It is possible that the antidepressant actions induced by i.p. administration of ARN-3236 were due to its peripheral SIK2 inhibition. To conclude out this possibility, ARN-3236 was stereotactically infused into the hippocampus of mice and a similar antidepressant efficacy was got, indicating that central SIK2 inhibition underlay the effects of ARN-3236. The western blotting, Co-IP and immunofluorescence results together suggested that ARN-3236 was able to prevent the stress-induced dysfunction in the hippocampal SIK2-CRTC1 system, BDNF signaling cascade and neurogenesis. These findings are consistent with our previous data involving SIK2-knockdown and SIK2-knockout, and further supporting the role of hippocampal SIK2 and CRTC1 in the pathophysiology of depression (Jiang et al., 2019). Besides to be a potential antidepressant, our findings suggest that ARN-3236 may also be a pro-neurotrophic/pro-neurogenic compound, especially as it has promoting actions on BDNF under normal condition. It has been well demonstrated that CRTC1, BDNF and neurogenesis play critical roles in not only depression but also many other neurological disorders, such as Alzheimer's disease, Parkinson's disease, stroke and epilepsy (Liu et al., 2010; Mu and Gage, 2011; Saura, 2012; Yu et al., 2013; Wang et al., 2016; Koh and Park, 2017; Parra-Damas et al., 2017; Tanila, 2017; Lim et al., 2018; Mohammadi et al., 2018). Thus, it is of great significance to investigate in the future whether ARN-3236 has beneficial effects on these disorders.

It is interesting that ARN-3236 treatment reduced the expression of SIK2. Normally, the inhibition of the enzymatic activity of a certain protein could lead to the increased expression of this protein because of the negative feedback mechanism. Here, it should be noticed that ARN-3236 treatment significantly enhanced the level of pCaMKIV, an important downstream signaling molecule of BDNF, while Sasaki et al. demonstrated that pCaMKIV was capable of phosphorylating SIK2 at Thr484 site, resulting in SIK2 degradation in cortical neurons (Sasaki et al., 2011). Thus, we have a speculation that ARN-3236 administration initially inhibits the activity of SIK2 but not influence its expression, while later, the ARN-3236-enhanced pCaMKIV causes SIK2 degradation, reducing its expression.

**FIGURE 13:**
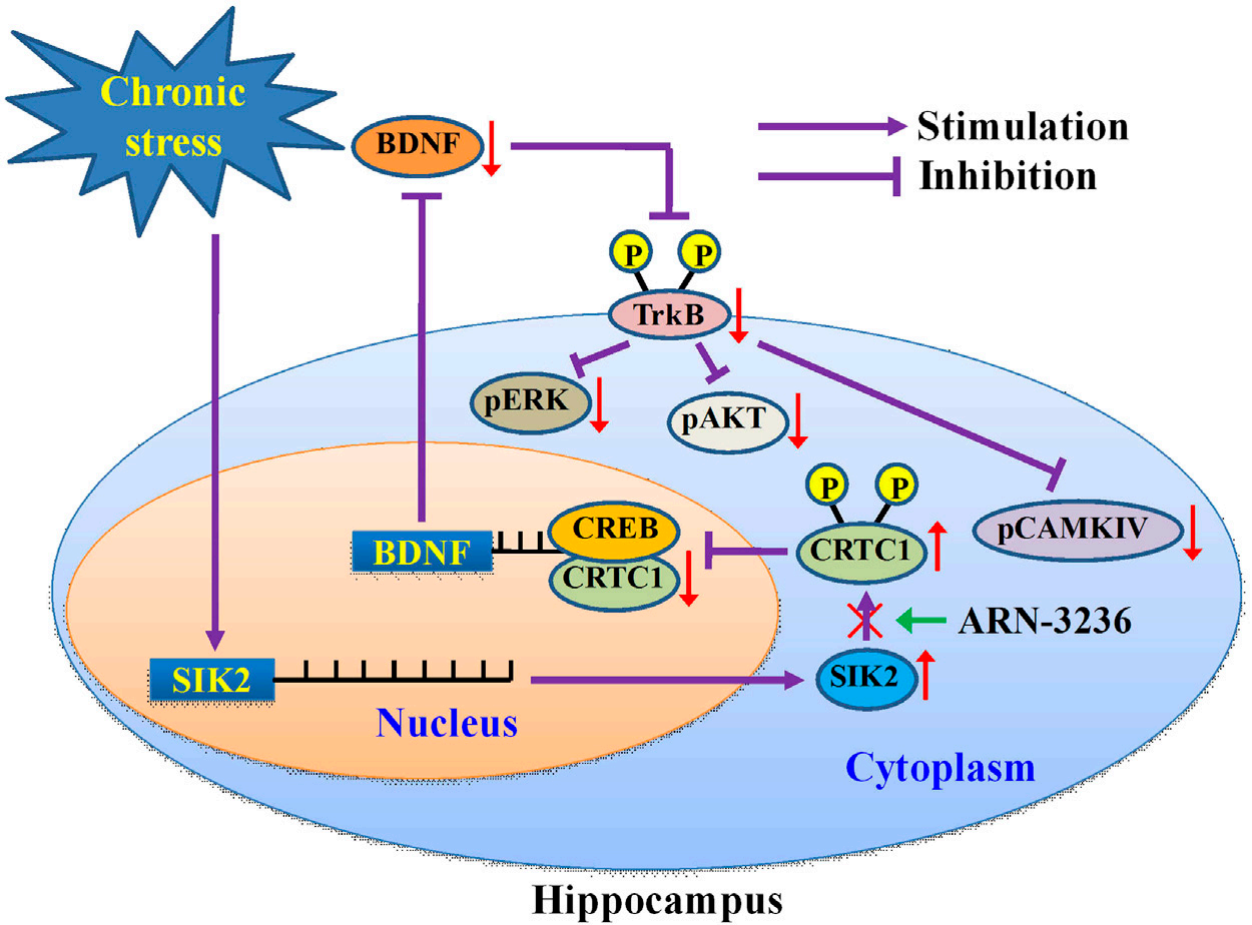
Schematic representation of a suggested model describing the antidepressant mechanism of ARN-3236. Chronic stress significantly promoted the expression of hippocampal SIK2, which phosphorylates cytoplasmic CRTC1 and prevents its nuclear translocation, leading to decreased CRTC1-CREB binding in the hippocampus. Due to the deficiency in CRTC1-CREB supporting, the levels of BDNF biosynthesis as well as its downstream signaling cascades (MAPK/ERK, PI3L/AKT and CaMK/CaMKIV pathways) are fully down-regulated, resulting in depression. ARN-3236 produces antidepressant-like actions by preventing the chronic stress-induced effects on the hippocampal SIK2-CRTC1-CREB-BDNF pathway.

The usage of CRTC1-shRNA, CREB-shRNA, BDNF-shRNA and TrkB-shRNA together confirmed that the hippocampal CRTC1-CREB-BDNF pathway is required for the antidepressant-like effects of ARN-3236 (Figure 13). In this study, BDNF was chosen as the downstream molecular target underlying the protecting effects of ARN-3236 against chronic stress, as BDNF is a very well-known member implicated in the pathogenesis of depression, and moreover, its biosynthesis is closely controlled by CRTC1 and CREB (Martinowich et al., 2007; Finsterwald et al., 2010; Esvald et al., 2020). However, the neurobiology of depression is very complex, involving not only BDNF but also a lot of other members regulated by CREB, such as mammalian target of rapamycin, vascular endothelial growth factor and peroxisome proliferator-activated receptor alpha (Clark-Raymond and Halaris, 2013; Abelaira et al., 2014; Song et al., 2018). SIK has also been demonstrated to modulate several other factors than CRTC, including polarity protein Par3, the Hippo signaling pathway and cytoplasmic histone deacetylase 4 (Wehr et al., 2013; Abend et al., 2017; Vanlandewijck et al., 2017). Therefore, for the pharmacological targets of ARN-3236, currently we could not yet exclude out these proteins mentioned above besides the CRTC1-CREB-BDNF pathway, and more shRNAs will be adopted in the future.

Indeed, the monoaminergic system is now still being targeted by drug companies in the search to find improved antidepressant agents with greater effectiveness and a faster onset of clinical action. A multitude of strategies and new promising compounds have been tested to potentiate monoaminergic neurotransmission. For example, one strategy is to target 5-HT receptors in combination with SERT inhibition in one molecule, and has led to two multimodal antidepressants (vilazodone and vortioxetine) which recently received market authorization for treating major depressive disorder (Dale et al., 2015). Besides, some other candidate drugs are currently under study, including triple reuptake inhibitors which simultaneously inhibit serotonin, noradrenaline and dopamine transporters (Amitifadine, LPM570065, etc.), as well as other molecules that act as agonists or antagonists at specific serotonergic receptors (Dale et al., 2015). Despite the pharmacological achievements in this field, the existing monoamine-based drugs fail to overcome the limitations of the more standard monoaminergic drugs. Other research efforts have focused on hypotheses of depression that go beyond the monoamines, which are based on modulation of glutamate neurotransmission, neuroplasticity, hypothalamic-pituitary-adrenal (HPA) axis, reward system, neuroinflammation and so on (Alamo and López-Muñoz, 2009; Vidal et al., 2011; Blier and El Mansari, 2013; Dale et al., 2015; Dean and Keshavan, 2017; Lima-Ojeda et al., 2018). There are a lot of compounds generated to test these theories, including NMDA receptor modulators (Lanicemine/AZD6765, Memantine, Traxoprodil/CP-101,606, GLYX-13/rapastinel, etc.), cortiocotropin releasing factor (CRF) antagonists (NBI-30775/R121919, CP-316,311), neurokinin (NK) antagonists (Aprepitant/MK869, L759274, Saredutant/SR48968, etc.), neuropeptide receptors antagonists (Filorexant/MK6096, ABT436), cyclooxygenase-2 (COX-2) inhibitors (celecoxib) and tumor necrosis factor (TNF) α antibody (Infliximab) (Dale et al., 2015). However, up to now, many of them have failed in the preclinical research due to various reasons. Here, our study offers a new antidepressant candidate, ARN-3236. So far there are only a few reports involving the pharmacological research of ARN-3236. For example, Zhou et al. showed that ARN-3236 could sensitize ovarian cell lines and xenografts to paclitaxel (Zhou et al., 2017). Lombardi et al. revealed that SIK inhibition in human myeloid cells by ARN-3236 modulated TLR and IL-1R signaling and induced an anti-inflammatory phenotype (Lombardi et al., 2016). Our findings not only extend the knowledge of ARN-3236’s pharmacological actions, but also further support that hippocampal SIK2 could be a novel target for antidepressant developments.

## Data Availability Statement

The original contributions presented in the study are included in the article/Supplementary Material, further inquiries can be directed to the corresponding authors.

## Ethics Statement

The animal study was reviewed and approved by Animal Welfare Committee of Nantong University.

## Author Contributions

BJ and JL designed this study. BJ wrote the manuscript. YL, WT, CJ, JG, and YC performed the experiments. XZ, YS, JH, CW, and WG helped collecting and analyzing the data. All authors read and approved this manuscript.

## Funding

This work was supported by a grant from the National Natural Science Foundation of China to BJ (No. 81873795), a grant from the Natural Science fund for colleges and universities in Jiangsu Province to BJ (No. 18KJB310013), and also a grant from the Science and Technology Projects of Nantong City to BJ (No. MS12018076).

## Conflict of Interest

The authors declare that the research was conducted in the absence of any commercial or financial relationships that could be construed as a potential conflict of interest.

## Abbreviations

AAV, adeno-associated virus; AKT, protein kinase B; ANOVA, analysis of variance; BDNF, brain derived neurotrophic factor; BBB, blood-brain barrier; Brdu, 5-Bromo-2-deoxyuridine; CaMKIV, Calcium/calmodulin-dependent protein kinase IV; Co-IP, Co-immunoprecipitation; CREB, cAMP response element-binding protein; CSDS, chronic social defeat stress; CUMS, chronic unpredictable mild stress; DCX, doublecortin; DG, dentate gyrus; ERK, extracellular regulated protein kinase; FST, forced swimming test; i.p., intraperitoneally; NeuN, Neuronal Nuclei; shRNA, short hairpin RNA; SIK, Salt-inducible kinase; TrkB, tyrosine kinase B; TST, tail suspension test
