# Supplementary material for: The Selective SIK2 Inhibitor ARN-3236 Produces Strong Antidepressant-Like Efficacy in Mice via the Hippocampal CRTC1-CREB-BDNF Pathway
Source: Front Pharmacol. 2021 Jan 14;11:624429. doi: 10.3389/fphar.2020.624429 (PMC7840484; doi:10.3389/fphar.2020.624429)
Supplement: Supplementary file 1 [file datasheet1.docx]

**Supplemental Information**

**Additional Methods and Materials**

*High performance liquid chromatography-tandem mass spectrometry (HPLC-MS)*

Naïve C57BL/6J mice (male, 8 weeks old, 22～24 g) received a single injection of 30 mg/kg ARN-3236, and were anesthetized using carbon dioxide and then sacrificed by cervical dislocation at 1 or 2 h after drug administration. The hippocampus tissues of each mouse were rapidly dissected after brain extraction, rinsed in 0.9% saline and then fully homogenized in 800 µl acetonitrile. The homogenates were subjected to centrifugation (12000 rpm × 15 min, room temperature) and the resulting supernatant was subjected to HPLC-MS analysis.

Quantification of ARN-3236 was done by HPLC-MS/MS using an Agilent 1260 HPLC system coupled to an AB SCIEX QTRAP 5500 tandem quadrupole mass spectrometer. The chromatographic system comprised a solvent manager, a sample manager and a column oven. The isocratic separation of ARN-3236 and the matrix was achieved with an Agilent ZORBAX SB-C18 (100 × 2.1 mm, 3.5 μm) reverse phase column maintained at 25°C. The mobile phase consisted of 20 mM ammonium formate solution (pH 4.0) and acetonitrile (*v/v* = 3:7) and the flow rate was 0.2 ml/min. The injection volume was 5 μL. ARN-3236 was analyzed using electrospray ionization (ESI: 337.1/321.8) in positive mode with the following source settings: ISV = 5500 V, TEM = 500 ºC, CUR = 35 psi, CAD = Medium, GS1 = 50 psi, GS2 = 50 psi, DP = 180 V, CE = 26 V and CXP = 20 V. The methods were validated with 3 replicates at 0.1, 2 and 20 ng/mL and showed good intra-day precision (RSD< 1%) and accuracy (> 91%).

*Stereotactic surgery and infusion*

Briefly, as we previously described (Song et al., 2018; Jiang et al., 2019; Wang et al., 2020), the experimental C57BL/6J mice were individually anesthetized (0.5% pentobarbital sodium, i.p., 50 mg/kg) and placed in stereotactic frames (Stoelting, Wood Dale, USA) when they showed muscle relaxation, slow corneal reflex and no skin pinch reaction. After exposing the skulls, double-guide cannulas (Plastics One, Roanoke, USA) were bilaterally implanted into the ventral hippocampus (AP = - 2.3 mm, ML = ± 1.6 mm, DV = + 1.8 mm) of each mouse, and fixed to the skull using dental cement. Each double-guide cannula was equipped with an inserted dummy cannula and a dust cap. Afterwards, the mice were given a 3-d recovery period under warm condition (31 ± 1°C) with good care.

For hippocampal infusion of ARN-3236, osmotic minipumps (ALZET®, Cupertino, USA) were designed to deliver 0.2 µl/min of ARN-3236/vehicle (1 µl/side). Each osmotic minipump was attached to a brain infusion cannula.

*Adenovirus associated virus (AAV)-mediated gene interference*

The hippocampal infusions of AAV-Control-shRNA-EGFP/AAV-CRTC1-shRNA-

EGFP/AAV-CREB-shRNA-EGFP/AAV-BDNF-shRNA-EGFP/AAV-TrkB-shRNA-EGFP into cannulas was performed bilaterally at a rate of 0.2 µl/min (1 µl/side) using 5 μL microsyringes (Hamilton, Fisher Labosi, France). The microsyringes were maintained in place for 4 min to limit reflux along the injection track and allow for virus diffusion. A 14-d period was required for AAV to spread over the whole hippocampus.

AAV-Control-shRNA-EGFP, AAV-CRTC1-shRNA-EGFP, AAV-CREB-shRNA-EG

FP, AAV-BDNF-shRNA-EGFP and AAV-TrkB-shRNA-EGFP were produced by Genechem Co., Ltd (Shanghai, China). The production procedures and shRNA sequences were described in our previous study (Jiang et al., 2019). All these virus were diluted to 5 × 10^12^ (genome copies/ml) using the enhanced infection solution (Genechem Co., Ltd, Shanghai, China).

*Western blotting*

After the behavioral testing, the experimental C57BL/6J mice were anesthetized using carbon dioxide and then sacrificed by cervical dislocation. The hippocampus tissues of each mouse were rapidly dissected after brain extraction and then homogenized in NP-40 lysis buffer (Beyotime, Shanghai, China) containing protease inhibitors (Roche, Basel, Switzerland). After proteins collection and denaturation, the expression of SIK1 (1:1000; Abcam, Bristol, UK; Cat# ab217809), SIK2 (1:1000; Cell Signaling, Danvers, USA), SIK3 (1:500; Abcam, Bristol, UK; Cat# ab88495), CRTC1 (1:1000; Abcam, Bristol, UK; Cat# ab92477), phospho-CRTC1-Ser151 (1:500; Merck Millipore, Billerica, USA; Cat# ABE560), BDNF (1:500; Abcam, Bristol, UK; Cat# ab108319), TrkB (1:1000; Abcam, Bristol, UK; Cat# ab187041), phospho-TrkB-Tyr515 (1:500; Thermo Fisher, Waltham, USA; Cat# PA5-36695), ERK1/2 (1:1000; Cell Signaling, Danvers, USA; Cat# 9102), phospho-ERK1/2-Thr202/Tyr204 (1:1000; Cell Signaling, Danvers, USA; Cat# 9101), AKT (1:1000; Cell Signaling, Danvers, USA; Cat# 9272), phospho-AKT-Ser473 (1:500; Cell Signaling, Danvers, USA; Cat# 4060), CaMKIV (1:1000; Abcam, Bristol, UK; Cat# ab3557), phospho-CaMKIV-Thr196/200 (1:1000; Abcam, Bristol, UK; Cat# ab59424), CREB (1:1000; Cell Signaling, Danvers, USA; Cat# 9197), phospho-CREB-Ser133 (1:500; Cell Signaling, Danvers, USA; Cat# 9198), Histone 2A (1:500; Cell Signaling, Danvers, USA; Cat# 2578) and β-actin (1:2000; Immunoway, Plano, USA; Cat# YT0099) proteins in the hippocampus were evaluated using the method which we have frequently reported (Jiang et al., 2017; Ren et al., 2017; Wang et al., 2017; Ni et al., 2018; Song et al., 2018; Xu et al., 2018; Jiang et al., 2019; Zhang et al., 2019; Wang et al., 2020). The Odyssey® Infrared Imaging system (Licor, Lincoln, USA) and ImageJ software (NIH, Bethesda, USA) were used together to do the analyses.

*Co-immunoprecipitation (Co-IP)*

After the behavioral testing, the experimental C57BL/6J mice were anesthetized and then sacrificed. The hippocampus tissues were dissected and homogenized. The protein supernatants were harvested and then the protein concentrations were determined. The detection of CRTC1-CREB protein binding in the hippocampus was performed using the method described in our previous study (Jiang et al., 2019). The protein A/G Plus-Agarose (Santa Cruz Biotech, Santa Cruz, USA), regular washing buffer (50 mM Tris-HCl, 100 mM NaCl, 1 mM EDTA, and 0.5% NP-40), high-salt washing buffer (50 mM Tris-HCl, 500 mM NaCl, 1 mM EDTA, and 0.5% NP-40), primary anti-CRTC1 (Abcam, Bristol, UK; Cat# ab92477) and anti-CREB (Cell Signaling, Danvers, USA; Cat# 9197) antibodies were employed in this method.

*Immunofluorescence (IF)*

Hippocampal doublecortin (DCX) staining was done as described before (Jiang et al., 2017; Wang et al., 2017; Song et al., 2018; Jiang et al., 2019; Zhang et al., 2019; Wang et al., 2020). In brief, the experimental C57BL/6J mice were anesthetized and then transcardially perfused with 0.1 M PBS followed by 4% paraformaldehyde (PFA). After post-fixing (4% PFA, 4°C, overnight) and dehydration (30% sucrose, 4°C, 2 d), 25 µm coronal sections were collected using a freezing microtome (Leica, Wetzlar, Germany). Free-floating sections were incubated in 0.3% Triton X-100 (30 min, Room temperature) and then 3% bovine serum albumin (BSA; 30 min, Room temperature), followed by incubation with primary anti-DCX antibody (1:100; Cell Signaling, Danvers, USA; Cat# 4604) overnight at 4°C. After three PBS washes, the sections were incubated with Fluorescein isothiocyanate (FITC)-conjugated secondary antibody (1:50; Thermo Fisher, Waltham, USA) for 2 h at room temperature. After three additional washes, the sections were incubated with DAPI (Beyotime, Shanghai, China) for 10 min at room temperature. Finally, the sections were washed again, coverslipped and visualized using a confocal laser scanning system (Leica, Wetzlar, Germany). Quantification of DCX immunolabeling in dentate gyrus (DG) has been described before in detail (Jiang et al., 2019).

Hippocampal NeuN/Brdu co-labeling was done as described before (Jiang et al., 2017; Wang et al., 2017; Song et al., 2018; Jiang et al., 2019; Zhang et al., 2019). Briefly, the experimental C57BL/6J mice were injected with Brdu (4 × 75 mg/kg, 2-h intervals/d) for 2 d and then perfused after 28 d. After post-fixing and dehydration, 25 µm coronal sections were collected and treated in the same way as above, except for no DAPI staining, different antibodies and three more steps at the beginning (1, incubation in 50% formamide/2 × SSC at 65°C for 2 h; 2, incubation in 2 N HCl at 37°C for 30 min; 3, incubation in 0.1 M boric acid buffer (pH 8.5) at room temperature for 10 min). The antibodies adopted were primary anti-Brdu (2 µg/ml, Roche, Basel, Switzerland; Cat# 11170376001), primary anti-NeuN (1:200; Abcam, Bristol, UK; Cat# 177487), FITC-conjugated and rhodamine-conjugated secondary antibodies (1:50; Thermo Fisher, Waltham, USA). The sections were visualized using a DM6B fluorescence microscope (Leica, Wetzlar, Germany). Quantification of NeuN/Brdu immunolabeling in the DG region has also been described before (Jiang et al., 2019).

**References**

Jiang, B., Wang, H., Wang, J.L., Wang, Y.J., Zhu, Q., Wang, C.N., et al., 2019. Hippocampal Salt-Inducible Kinase 2 Plays a Role in Depression via the CREB-Regulated Transcription Coactivator 1-cAMP Response Element Binding-Brain-Derived Neurotrophic Factor Pathway. Biol. Psychiatry 85, 650-666.

Jiang, B., Wang, Y.J., Wang, H., Song, L., Huang, C., Zhu, Q., et al., 2017. Antidepressant-like effects of fenofibrate in mice via the hippocampal brain-derived neurotrophic factor signalling pathway. Br. J. Pharmacol. 174, 177-194.

Ni, Y.F., Wang, H., Gu, Q.Y., Wang, F.Y., Wang, Y.J., Wang, J.L., et al., 2018. Gemfibrozil has antidepressant effects in mice: Involvement of the hippocampal brain-derived neurotrophic factor system. J. Psychopharmacol. 32, 469-481.

Ren, Y., Wang, J.L., Zhang, X., Wang, H., Ye, Y., Song, L., et al., 2017. Antidepressant-like effects of ginsenoside Rg2 in a chronic mild stress model of depression. Brain Res. Bull. 134, 211-219.

Song, L., Wang, H., Wang, Y.J., Wang, J.L., Zhu, Q., Wu, F., et al., 2018. Hippocampal PPARα is a novel therapeutic target for depression and mediates the antidepressant actions of fluoxetine in mice. Br. J. Pharmacol. 175, 2968-2987.

Wang, C.N., Gong, S.N., Guan, W., Wang, J.L., Gao, T.T., Wang, Y., et al., 2020. Hippocampal overexpression of chordin protects against the chronic social defeat stress-induced depressive-like effects in mice. Brain Res. Bull. 158, 31-39.

Wang, H., Zhao, Y., Wang, Y.J., Song, L., Wang, J.L., Huang, C., et al., 2017. Antidepressant-like effects of tetrahydroxystilbene glucoside in mice: Involvement of BDNF signaling cascade in the hippocampus. CNS Neurosci. Ther. 23, 627-636.

Xu, D., Sun, Y., Wang, C., Wang, H., Wang, Y., Zhao, W., et al., 2018. Hippocampal mTOR signaling is required for the antidepressant effects of paroxetine. Neuropharmacology 128, 181-195.

Zhang, J.J., Gao, T.T., Wang, Y., Wang, J.L., Guan, W., Wang, Y.J., et al., 2019. Andrographolide Exerts Significant Antidepressant-Like Effects Involving the Hippocampal BDNF System in Mice. Int. J. Neuropsychopharmacol. 22, 585-600.

**Supplemental Figures**

**
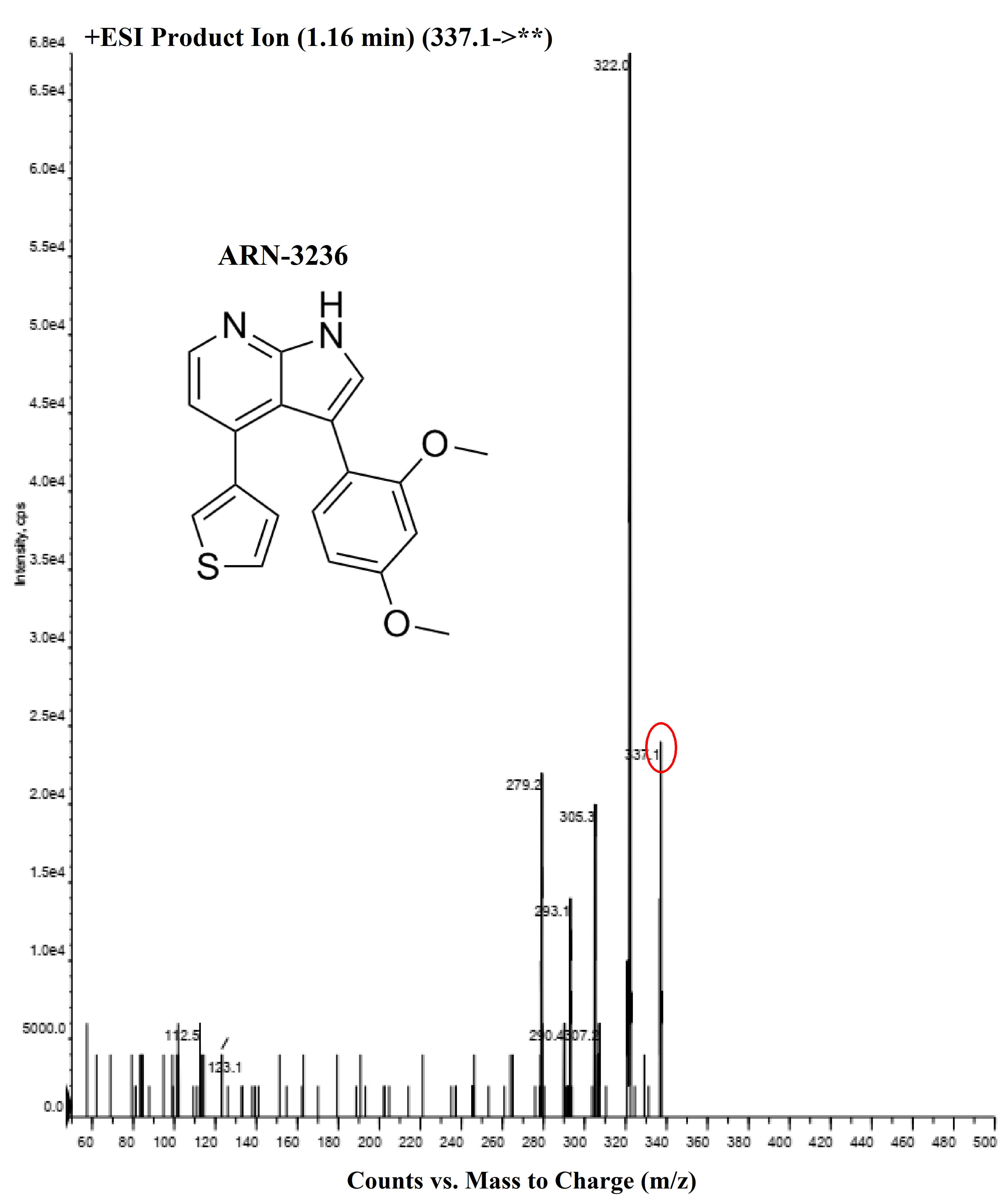
**

**Figure S1.** The chemical structure of ARN-3236 and mass spectra of ARN-3236 in the hippocampus tissues of mice.

**
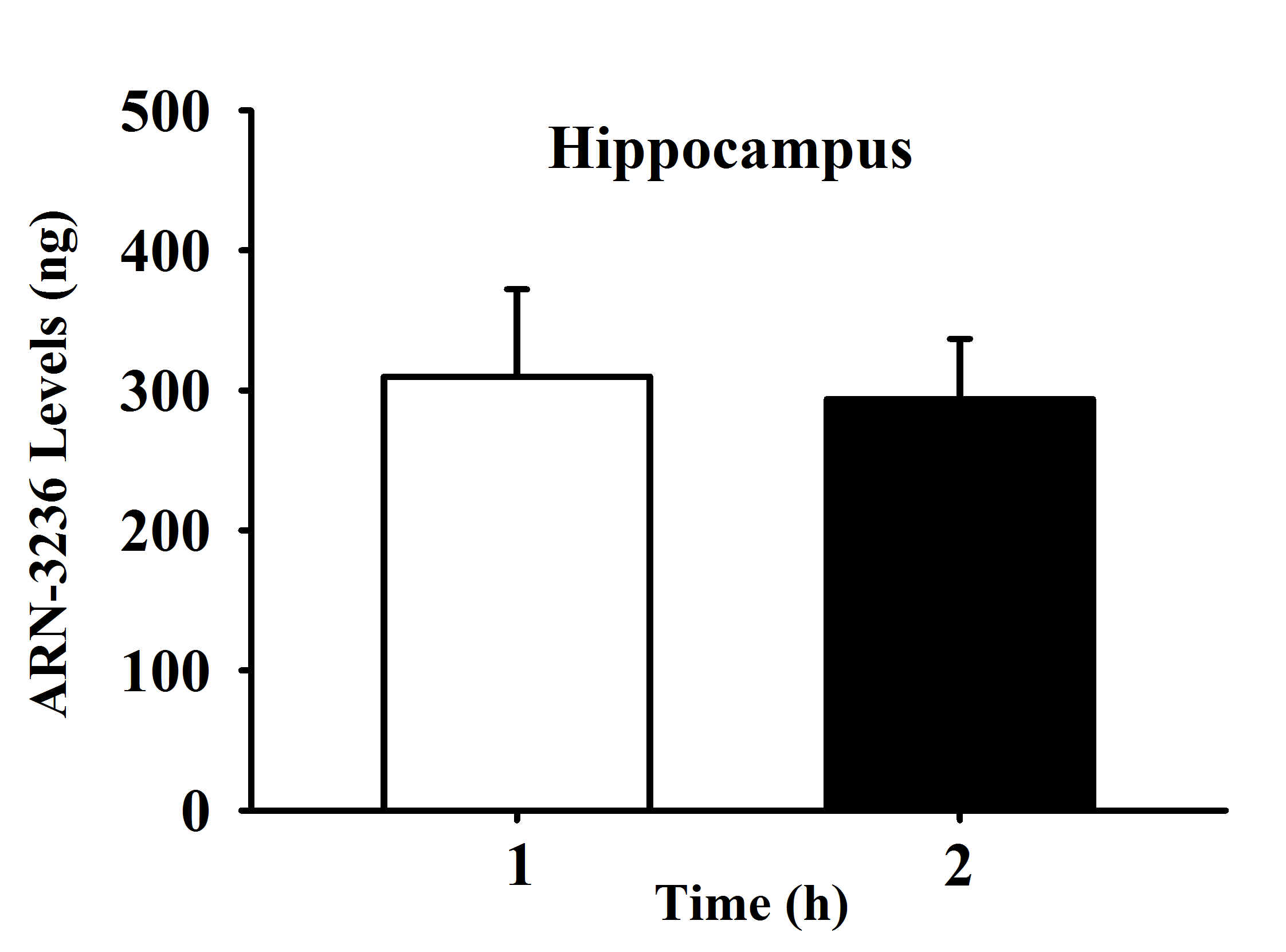
**

**Figure S2.** ARN-3236 has distributions in the hippocampus of mice at 1 and 2 h after a single administration of 30 mg/kg ARN-3236. All results are expressed as means ± S.E.M; n = 12 for 1 h, n = 11 for 2 h.
